# No signs of neurodegenerative effects in 15q11.2 BP1-BP2 copy number variant carriers in the UK Biobank

**DOI:** 10.1038/s41398-023-02358-w

**Published:** 2023-02-18

**Authors:** Rune Boen, Tobias Kaufmann, Oleksandr Frei, Dennis van der Meer, Srdjan Djurovic, Ole A. Andreassen, Kaja K. Selmer, Dag Alnæs, Ida E. Sønderby

**Affiliations:** 1 Department of Medical Genetics, Oslo University Hospital, Oslo, Norway; 2 NORMENT, Division of Mental Health and Addiction, Oslo University Hospital and Institute of Clinical Medicine, University of Oslo, Oslo, Norway; 3 Department of Psychiatry and Psychotherapy, Tübingen Center for Mental Health, University of Tübingen, Tübingen, Germany; 4 Centre for Bioinformatics, Department of Informatics, University of Oslo, Oslo, Norway; 5 School of Mental Health and Neuroscience, Faculty of Health, Medicine and Life Sciences, Maastricht University, Maastricht, The Netherlands; 6 NORMENT, Department of Clinical Science, University of Bergen, Bergen, Norway; 7 KG Jebsen Centre for Neurodevelopmental Disorders, University of Oslo, Oslo, Norway; 8 Department of Research and Innovation, Division of Clinical Neuroscience, Oslo University Hospital and the University of Oslo, Oslo, Norway; 9 Kristiania University College, Oslo, Norway

**Keywords:** Medical genetics, Neuroscience, Biomarkers

## Abstract

The 15q11.2 BP1-BP2 copy number variant (CNV) is associated with altered brain morphology and risk for atypical development, including increased risk for schizophrenia and learning difficulties for the deletion. However, it is still unclear whether differences in brain morphology are associated with neurodevelopmental or neurodegenerative processes. This study derived morphological brain MRI measures in 15q11.2 BP1-BP2 deletion (*n* = 124) and duplication carriers (*n* = 142), and matched deletion-controls (*n* = 496) and duplication-controls (*n* = 568) from the UK Biobank study to investigate the association with brain morphology and estimates of brain ageing. Further, we examined the ageing trajectory of age-affected measures (i.e., cortical thickness, surface area, subcortical volume, reaction time, hand grip strength, lung function, and blood pressure) in 15q11.2 BP1-BP2 CNV carriers compared to non-carriers. In this ageing population, the results from the machine learning models showed that the estimated brain age gaps did not differ between the 15q11.2 BP1-BP2 CNV carriers and non-carriers, despite deletion carriers displaying thicker cortex and lower subcortical volume compared to the deletion-controls and duplication carriers, and lower surface area compared to the deletion-controls. Likewise, the 15q11.2 BP1-BP2 CNV carriers did not deviate from the ageing trajectory on any of the age-affected measures examined compared to non-carriers. Despite altered brain morphology in 15q11.2 BP1-BP2 CNV carriers, the results did not show any clear signs of apparent altered ageing in brain structure, nor in motor, lung or heart function. The results do not indicate neurodegenerative effects in 15q11.2 BP1-BP2 CNV carriers.

## Introduction

The human brain undergoes structural changes as people age, including cortical thinning and volume loss [1–4]. Such age-related changes in brain morphology have been linked to cognitive decline [5, 6] and physical deterioration [7], and are presumably the results of a complex interplay between many neurobiological processes, including links to genetic factors [8–10]. Ageing affects individuals differently as reflected in increased heterogeneity in brain structure and cognition in mid to late adulthood [11]. In the last couple of decades, several potential age biomarkers have been investigated, e.g., through the use of measures of telomere length, DNA methylation [12–14] and brain structure.

Studies have used machine learning (ML) techniques to predict chronological age by using structural MRI measures as input features [15–17]. The estimations provide a measure of an individual’s brain age gap, i.e., the difference between the predicted age and chronological age. Higher brain age gap has been suggested to reflect either (i) an accelerated ageing rate throughout life, (ii) an accentuated, but stable ageing, or iii) an accentuated and accelerated ageing [12]. Higher predicted brain age has been associated with other indicators of older age—e.g. accelerated body age, older physical appearance, lower cognitive functioning [15], weaker grip strength, poorer lung function, slower walking speed, and increased mortality risk [18]. Brain age gap has been found to be heritable and increased in several neurological and psychiatric conditions [17, 19], linking brain age to brain disorders and genetics. However, studies investigating brain age in individuals carrying rare genetic variants and displaying alterations in brain structure are scarce.

Some rare recurrent copy number variants (CNVs), i.e., regions of the genome that are either deleted or duplicated, are associated with alterations in brain structure [20–23]. Individuals carrying recurrent microdeletions or duplications at the 15q11.2 BP1-BP2 genomic locus display differences in brain morphology through altered cortical thickness, surface area, and white matter fiber tracts [24, 25]. The 15q11.2 BP1-BP2 genomic region contains four evolutionary highly conserved genes: *CYFIP1*, *TUBGCP5*, *NIPA1,* and *NIPA2* [26], which have been reported to be critical for typical neurodevelopment [27]. Indeed, both the *CYFIP1* and the *NIPA1* are highly expressed in the developing mouse brain [28]. Further, the *CYFIP1* has been suggested to important for dendritic spine morphology [29, 30] and myelination [31]. The *NIPA1* and the *NIPA2* encode for magnesium transporters [32], whereas the *TUBGCP5* is important for microtubule nucleation [33]. At the phenotypic level, 15q11.2 BP1-BP2 deletion carriers have an increased risk for schizophrenia [34] and learning difficulties, such as dyslexia and dyscalculia [35], as well as lower cognitive functioning [24, 25]. Duplication carriers, on the other hand, have not been convincingly associated with any neurodevelopmental or psychiatric disease and perform similarly to non-carriers on cognitive tests [35–37]. Cortical thickness displays a dose response of the 15q11.2 BP1-BP2 CNVs (i.e., decreased cortical thickness with increasing copy number) with deletion carriers having significantly thicker cortices, lower surface area and smaller nucleus accumbens compared to non-carriers. Cortical thickness, surface area, and subcortical volume are structural brain measures that exhibit clear age-related changes during typical aging, including cortical thinning [9, 38, 39], reductions in surface area [40] and lower subcortical volume [41]. However, it is unclear whether the group differences in brain structure among 15q11.2 BP1-BP2 CNV carriers are reflected by altered ageing as indicated by estimations of brain age gap. There are also other factors that may indicate accentuated ageing among 15q11.2 BP1-BP2 CNV carriers. For instance, the 15q11.2 BP1-BP2 CNV is also associated with several physical traits that typically deteriorate in older ages, including reaction time, hand grip strength, lung function and blood pressure [25, 42]. All of these measures are important features for an age biomarker that is associated with mortality and hospital admissions in older individuals [43], emphasizing their clinical significance. In sum, the differences in brain morphology and performance in physical traits that declines with age may indicate altered ageing among 15q11.2 BP1-BP2 CNV carriers.

In the current study, we investigated apparent brain ageing in 15q11.2 BP1-BP2 CNV carriers. Due to alterations in brain morphology and in physical traits among 15q11.2 BP1-BP2 CNV carriers [25, 42], i.e., where the majority of these traits indicate accentuated ageing, we predicted that the 15q11.2 BP1-BP2 CNV carriers would also exhibit group differences in brain age gap. To test for accelerated or decelerating ageing in other age-affected measures, we follow-up brain age gap estimations with testing for a cross-sectional interaction effect between 15q11.2 BP-BP2 copy number and age on brain measures and an effect on the longitudinal ageing trajectory in reaction time, grip strength, lung function and blood pressure.

## Methods

### Participants

The present study includes 124 15q11.2 BP1-BP2 deletion and 142 duplication carriers, 496 non-carrier deletion controls and 568 non-carrier duplication controls from the UK biobank study—all with neuroimaging data. Four non-carrier controls were matched to each CNV carrier based on age, sex, scanner site, affection status (diagnosis of a neurological or mental/behavioral disorder) and estimated intracranial volume (ICV). The CNVs were identified as described previously [25]. We also extracted a sample of individuals diagnosed with multiple sclerosis (*n* = 60) to validate the brain age prediction models against the unaffected participants from the deletion-control and duplication-control groups (*n* = 1210). A training group consisting of non-carrier individuals (*n* = 36,013 individuals) with no reported neurological or mental/behavioral diagnoses was used as a training set for brain age prediction (see supplementary note 1 and supplementary table 1 for details and descriptive statistics for the full data set). The estimated effect sizes for group differences that could be reliably detected using the current test sample ranged from Cohen’s *d* = 0.26 to 0.36 (see supplementary note 2 for power sensitivity analysis).

### MRI acquisition

All MRI data were acquired on a 3 T Siemens Skyra scanner from three scanner sites. Detailed information about the image processing and quality control for the UK biobank is reported elsewhere [44]. Briefly, the T1-weighted images were acquired with a sagittal orientation at 1.0 mm isotropic resolution, TI = 880 ms, TE = 2.01 ms, TR = 2000 ms.

### Structural brain/morphology measures

The MR images were preprocessed in Freesurfer version 5.3.0 [45] and provided estimates of cortical thickness, surface area, volume across 180 regions per hemisphere, in addition to subcortical and cortical summary measures using the Human Connectome Project (HCP) parcellation atlas [46]. These HCP features were used to create models for brain age prediction in a training set and used to predict brain age in an independent test sample (see below). Participants were excluded if they had a Euler number that was missing or exceeded three standard deviations from the sample mean, or if they had mean values that exceeded four standard deviations from the sample mean on cortical thickness, surface area or subcortical volume adjusted for covariates (see supplementary notes 1 for details). Here, we focus on the mean cortical thickness, total cortical surface area, and total subcortical volume to increase statistical power. Regional group differences between 15q11.2 BP1-BP2 CNV carriers and non-carriers have been reported elsewhere for 34 regions of interest [25]. However, to fully exploit the complex relationship between brain structures, we used all of the values from the parcellated brain regions to get a single estimated score for each participant (i.e., predicted age).

### Motor, lung, and heart function

Reaction time, grip strength, forced expiratory volume (lung function), systolic and diastolic blood pressure (incl. body mass index (BMI) as covariate for measures of blood pressure) were extracted from the UK biobank. These measures are available from up to three timepoints, resulting in a mix of cross sectional and longitudinal data. We used mixed effects models in these analyses to cope with the dependency in the data.

### Brain age gap estimations

We used the XGBoost package in R [47, 48] to build a ML model to predict age from a set of cortical thickness, cortical surface area, cortical volume, subcortical volume (i.e., 180/180/180/8 regions of interest for each hemisphere) and cortical summary measures from the training group, in total 1145 measures (similar to; [17, 49, 50] henceforth termed the ‘full ML model’). In addition, we trained three separate ML models that included either measures of (i) cortical thickness (360 measures), (ii) surface area (360 measures) or (iii) subcortical volume measures (16 measures) only to predict age. These three models will be referred to as ‘cortical thickness ML model’, ‘surface area ML model’ and ‘subcortical volume ML model’, respectively. The XGBoost package was used as it is resource efficient and flexible, including implementation of machine learning algorithms using gradient boosting, parallel computation, and flexible parameter settings. It has also been shown to be superior to other machine learning models as demonstrated in machine learning competitions [47]. Parameters for the brain age ML models were tuned individually for each model following an optimization procedure (see supplementary note 3 for details). An overestimation and underestimation of the predicted ages at the tails of the chronological age distribution is commonly observed in brain age prediction models [49, 51, 52]. Thus, we corrected for this bias using a recent correction method for predicted brain age [53] (see supplementary note 4 for details). Next, we subtracted each individual’s chronological age from the corrected predicted brain age to get an estimate of brain age gap. That is, if an individual is 50 years of age, while the predicted brain age is 52, the 2-year brain age gap will indicate that the individual has an older looking brain than what is expected based on the individual’s chronological age.

### Statistical analysis

All analyses were carried out in R version 4.0.0. The brain measures (i.e. mean cortical thickness, total cortical surface area, total subcortical volume, brain age gap) were pre-residualized for age, age^2^, sex, scanner site, affection status, ICV and Euler number using linear regression. First, for group comparisons, deletion and duplication carriers were compared to their respective non-carrier matched control group, and deletion carriers were compared to duplication carriers. The dependent variables were brain morphological measures (mean cortical thickness, total cortical surface area, total subcortical volume) and brain age gap (i.e., derived from the ‘cortical thickness ML model’, ‘surface area ML model’, ‘subcortical volume ML model’, and the ‘full ML model’). These were tested using a two-sided t-test. Cohen’s d was calculated as measure of effect size for the group comparisons.

Second, we investigated an interaction effect between age and carrier status using the following linear model: Y ~ age + age^2^ + carrier status + age*carrier status after regressing out the effect of scanner site, affection status, ICV and Euler number from the dependent variables. Carrier status was coded as “yes” and “no” (i.e., “yes” for deletion and duplication carriers, and “no” for deletion-control and duplication-control). The interaction effect was tested separately between the 15q11.2 BP1-BP2 CNV groups, such that deletion carriers and deletion-control were tested against each other, and duplication-carriers and duplication control were tested against each other. Dependent variables were mean cortical thickness, total cortical surface area and total subcortical volume.

Third, we examined the age-related trajectory of reaction time, grip strength, lung function, systolic and diastolic blood pressure. We used cross sectional and longitudinal data (First visit: *n* = 1330, Mean age = 55.3, SD = 7.53, age range = 40.5–70.6, Second visit: *n* = 224, Mean age = 60.8, SD = 7.07, age range = 45.4–73.8, Third visit: *n* = 1330, Mean age = 64.2, SD = 7.62, age range = 46.8–81.3) and mixed effects models. The models were fitted with a random effect of participant on intercepts and with sex and affection status included as covariates (+BMI for the blood pressure measures). To obtain the best ageing model, we compared models with either: (a) only covariates, (b) age and covariates, or (c) age, age^2^ and covariates. The models were tested stepwise to get the age model that best fitted the data. Then, we tested this model against the same model but including carrier status as either (d) main effect or (e) interaction effect. We used the Akaike information criterion (AIC) as model criterion, where the more complex model was chosen if the AIC dropped by 2 with a *p*-value < 0.05 (see supplementary note 5 for details). Finally, we examined the correlation between brain age gap and age-affected physical measures (reaction time, grip strength, lung function, systolic and diastolic blood pressure). Here, we pre-residualized the measures for the variables included in the model that best fit the data as described above, before running the correlations. Bonferroni corrections were conducted across all comparisons. The significance threshold for group comparisons on measures of brain structure and brain age gap (21 comparisons), interaction effect between age and measures of brain structure (6 comparisons), and brain age gap correlations with measures of motor, lung, and heart function (10 correlations), resulted in alpha set to 0.05/ (21 + 6 + 10) = 0.0014. For the models of motor, lung, and heart function, the predictors were considered significant if the *p* < 0.05 in the model that best fitted the data according to the selection criterions stated above. The uncorrected *p*-value is presented across all analyses.

## Results

### 15q11.2 copy number variants and brain morphology

**Fig. 1:**
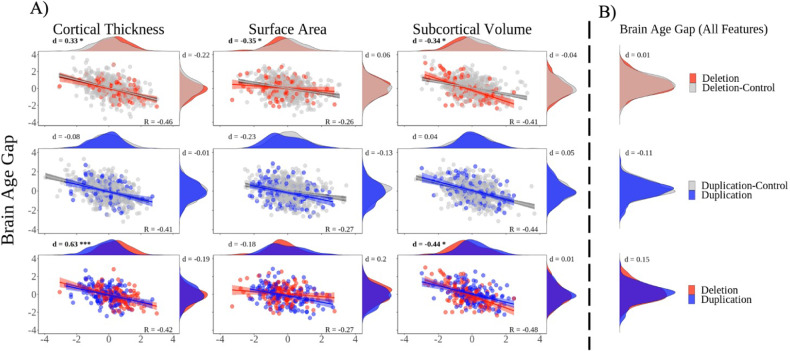
Brain morphology and brain age in 15q11.2 BP1-BP2 carriers. **A** Scatterplots: Residualized brain measures vs brain age gap. Density plots; Horizontal: Brain measures, Vertical: Brain age gaps from the ‘cortical thickness machine learning model’, ‘surface area machine learning model’, and ‘subcortical volume machine learning model’. Columns: Mean cortical thickness (left), total surface area (middle), and total subcortical volume (right). Rows: Comparisons between: Deletion carriers versus deletion-controls, duplication carriers versus duplication-controls and deletion carriers versus duplication carriers. **B** Brain age gap group differences obtained from the ‘full machine learning model’. All values were adjusted for age, age^2^, sex, scanner site, affection status (having either a F or G- ICD10 diagnosis), ICV and Euler number. All values were scaled for visualization purposes. Raw residuals, confidence intervals, and *p*-values can be found in Supplementary Figs. 1–3 (for measures of brain morphology) and Supplementary Figs. 6–9 (for measures of brain age gap). Red = deletion, carriers, gray = non-carriers, blue = duplication-carriers. *R* = correlation value across the two included groups. *d* = Cohens *d*. Group differences that survive the multiple comparison threshold are marked in bold. *p < 0.0014, ***p < 0.000014.

In the current study, 15q11.2 BP1-BP2 deletion carriers had significantly thicker cortex (*d* = 0.33, CI = 0.13, 0.53; *d* = 0.63, CI = 0.38, 0.87, both *p* < 0.001), and lower subcortical volume (*d* = −0.34, CI = −0.54, −0.14; *d* = −0.44, CI = −0.68, −0.20, both *p* < 0.001) compared to deletion-controls and duplication carriers, respectively, and lower total surface area (*d* = −0.35, CI = −0.55, −0.15, *p* < 0.001) in comparison to deletion-controls (Fig. 1; Supplementary Figs. 1–3). The results did not reveal any significant difference for duplication-carriers compared to duplication-controls, nor any interaction effects of age and carrier status on any of the brain measures (see Supplementary Tables 3–4; Supplementary Fig. 4). Part of the sample has previously shown higher mean cortical thickness and lower total cortical surface area in deletion carriers compared to non-carriers, and lower mean cortical thickness in duplication carriers compared to non-carriers [25].

### Brain age gap in 15q11.2 BP1-BP2 copy number variant carriers

The ‘full ML model’ yielded the highest correlation between predicted age and chronological age (*r* = .80) outperforming the three simpler/narrower models—the ‘cortical thickness ML model’ (*r* = .71), the ‘surface area ML model’ (*r* = 0.63), and the ‘subcortical volume ML model’ (*r* = 0.64; Supplementary Fig. 5). None of the four ML models for brain age gap estimations showed any significant differences between 15q11.2 BP1-BP2 deletion and duplication carriers and non-carriers, nor between deletion and duplication carriers (Fig. 1A, B; Supplementary Figs. 6–9). Overall, brain age gap from the cortical thickness, surface area or subcortical volume ML models correlated negatively with the mean cortical thickness, total surface area and total subcortical volume, respectively (*r* ranging from −0.26 to −0.48; Fig. 1A). To ensure that the lack of identified brain age gap was not a result of poor brain age models, we briefly validated our brain age models in a sample of individuals diagnosed with multiple sclerosis. In support of the validity of our models, the results showed strong group differences in brain age gap between the multiple sclerosis group and a healthy control group Cohens *d* = 0.49 to 1.11; see Supplementary Table 6), which is in line with the previous literature [17, 54]. Thus, this leads us to the conclusion that the 15q11.2 BP1-BP2 CNV is not associated with a clear altered brain age gap.

### Age-related decline in motor, lung, and heart function

None of the mixed models that included an interaction term between age and carrier status yielded a better model fit compared to the other age models without the inclusion of the interaction term. Thus, generally the 15q11.2 BP1-BP2 deletion and duplication carriers do not deviate strongly from non-carriers on the expected age-related reductions in reaction time, grip strength, lung function, and diastolic and systolic blood pressure based on mixed cross-sectional and longitudinal data (Supplementary Fig. 10; Supplementary Tables 6–7). However, there were some main effects of carrier status, as the deletion carriers had slower reaction time compared to deletion-controls (Supplementary Table 6), whereas duplication carriers exhibited higher diastolic and systolic blood pressure compared to duplication-controls (Supplementary Table 7). In general, there were no significant associations between brain age gap derived from the ‘full ML model’ and the selected age-affected physical measures among the deletion-carriers and deletion-controls or duplication-carriers and duplication-controls. The exception was for the duplication-carriers and duplication-controls, that displayed a significant negative correlation between higher brain age gap and lower lung function (*r* = −0.17, *p* < 0.001; Supplementary note 6).

## Discussion

Focusing exclusively on an ageing population (47–81 years of age), we showed that the 15q11.2 BP1-BP2 deletion carriers did not display a significant difference in brain age gap in comparison to non-carriers. In concordance with this pattern, the cross-sectional age effect on cortical thickness, surface area and subcortical volume did not significantly interact with 15q11.2 BP1-BP2 copy number, indicating comparable ageing trajectory in brain structure. Likewise, the mixed cross-sectional and longitudinal analysis indicate that both deletion and duplication-carriers had age-related deterioration at a comparable rate to non-carriers in selected age-affected measures—reaction time, grip strength, lung function and blood pressure. Further, we expanded on previous results to show that 15q11.2 BP1-BP2 deletion carriers exhibited a lower total subcortical volume overall compared to deletion-control and duplication-carriers and confirmed results on cortical thickness and surface area (i.e., thicker cortex and lower surface area in deletion carriers compared to non-carriers). Thus, the data did not show evidence for accelerated ageing among 15q11.2 BP1-BP2 CNV carriers compared to non-carriers but rather a stable ageing trajectory. Brain age gap—the difference between the predicted age and chronological age—has been suggested to reflect brain ageing, where higher brain age gap may reflect accelerated ageing, or accentuated, but stable ageing, or accentuated and accelerated ageing [12]. In contrast to our hypothesis, the current study shows that group differences in brain structure among 15q11.2 deletion carriers did not coincide with group differences in brain age gap estimations, neither when using a ML model with many (i.e., ‘the full ML model’) or fewer features (i.e., ‘cortical thickness ML model’, ‘surface area ML model’, or ‘subcortical volume ML model’). The results indicate stable adult group-level differences in brain structure between the 15q11.2 BP1-BP2 deletion carriers and non-carriers. This seems to indicate that the observed group differences in brain phenotypes have been established before the individuals reached their current age range. This would indicate that the structural differences are due to other factors unrelated to ageing. Whilst caution is warranted given the lack of data to test this more directly, this may suggest that the brain alterations identified in 15q11.2 BP1-BP2 CNV carriers are more likely explained by an early offset in brain structure caused by early atypical neurodevelopment rather than neurodegeneration.

This study has strengths and limitations. The cross-sectional nature of the MR data limits our interpretation of the brain ageing trajectory. For instance, it is crucial to follow individuals over time to gain information on the slope of brain atrophy, ideally through multiple time points across the lifespan. As always, caution should be made when interpreting the results of cross-sectional data. Future studies using longitudinal brain imaging data are needed to fully characterize the age-related changes in brain structure. However, our mixed cross-sectional and longitudinal data on other age-affected measures (i.e., motor, lung and heart function) do seem to indicate a similar ageing trajectory between 15q11.2 BP1-BP2 CNV carriers and non-carriers.

The interpretation of the brain age gap estimations from the brain age models are relying on the assumption that the models are reliable and valid. Indeed, the brain age gap is simply the difference between the predicted chronological age and the chronological age, which means that the brain age gap is the error term from the brain age model. In the current study, however, brain age gap was associated with lung function among a sample of 15q11.2 BP1-BP2 duplication carriers and duplication-controls. This indicates that the brain age gap also carries useful biological information beyond measurement error. In addition, the current study showed that our brain age model yielded strong group differences between a multiple sclerosis group and a healthy control group, replicating previously identified brain age gap differences [17, 54]. Furthermore, brain age estimations are based on models that are informed by the strong associations between MRI features and age. Thus, deviation from a healthy brain age trajectory might reflect alterations in brain structure unrelated to ageing. It has also been suggested that brain age might reflect variations in the brain from early life [15]. Indeed, if the brain age models were simply reflecting variations in the brain, we would expect the 15q11.2 BP1-BP2 CNV carriers to exhibit alterations in brain age as they exhibit group-level differences in cortical thickness, surface area, and subcortical volume. However, we did not find any significant group differences in brain age gap among 15q11.2 BP1-BP2 CNV carriers. The low prevalence of carriers with the 15q11.2 BP1-BP2 CNV hinders the detection of small effects. Thus, the lack of significant statistical group differences does not imply that there is no neurodegenerative effect at all. However, the results do not support strong neurodegenerative effects that would have been of potential clinical relevance.

The 15q11.2 BP1-BP2 CNV carriers show phenotypic heterogeneity [25, 28, 35, 55]. This is important to note as the sample is drawn from the UK Biobank, which has a healthy volunteer bias at baseline [56], as well as a bias in the follow-up components [57]. For instance, the participants in the UK Biobank have been found to be healthier (e.g., lower rate of cancer, lower levels of smoking and daily alcohol consumption, lower likelihood of obesity) [56]. In addition, the follow-up components in the UK Biobank have been shown to be influenced by sample characteristics, including cognitive ability, adiposity, and liability to certain neurodevelopmental disorders [57]. This could potentially underestimate differences between 15q11.2 BP1-BP2 carriers and non-carriers. Despite the overall biases in the UK Biobank, we still identify group-level differences in brain structure but not in their brain age gap. Thus, these results do not support clear neurodegenerative effects among the 15q11.2 BP1-BP2 CNV. However, more research is needed in an unbiased population and caution is urged if extrapolating the current result to the full population of 15q11.2 BP1-BP2 carriers.

## Conclusion

To conclude, the 15q11.2 BP1-BP2 deletion carriers exhibit altered cortical thickness, surface area and subcortical volume compared to non-carriers. We did not find support for the hypothesis that the differences in brain structure among 15q11.2 BP1-BP2 CNV carriers are due to accentuated ageing neither in cross-sectional MR data, brain age gap, or age-affected physical measures. Thus, despite altered brain morphology and worse performance in physical traits, these deviations do not seem to have significant clinical implications for neurodegeneration or physical deterioration in an ageing sample. The altered brain morphology in 15q11.2 BP1-BP2 CNV carriers could reflect other factors unrelated to ageing, possibly atypical neurodevelopment. Future studies should investigate early developmental trajectories of 15q11.2 BP1-BP2 CNV carriers on brain structure and other physical measures to clarify the life-span trajectory of the altered brain morphology. Finally, the “healthy volunteer” bias in the UK Biobank warrants caution when interpreting the results, and studies examining age-related changes in a more population-representative sample are needed.

## Supplementary information

Supplementary Materials

## References

[1] Fjell AM, McEvoy L, Holland D, Dale AM, Walhovd KB (2014). What is normal in normal aging? Effects of aging, amyloid and Alzheimer’s disease on the cerebral cortex and the hippocampus. Prog Neurobiol.

[2] Fjell AM, Westlye LT, Amlien I, Espeseth T, Reinvang I, Raz N (2009). High consistency of regional cortical thinning in aging across multiple samples. Cereb Cortex.

[3] Fjell AM, Walhovd KB (2010). Structural brain changes in aging: courses, causes and cognitive consequences. Rev Neurosci.

[4] Frangou S, Modabbernia A, Williams SCR, Papachristou E, Doucet GE, Agartz I, et al. Cortical thickness across the lifespan: Data from 17,075 healthy individuals aged 3–90 years. Hum Brain Mapp. 2021. [cited 2021 Oct 25] Available from: https://onlinelibrary.wiley.com/doi/abs/10.1002/hbm.25364. 10.1002/hbm.25364 PMC8675431 33595143

[5] Persson J, Pudas S, Lind J, Kauppi K, Nilsson LG, Nyberg L (2012). Longitudinal structure-function correlates in elderly reveal MTL dysfunction with cognitive decline. Cereb Cortex.

[6] Fjell AM, Westlye LT, Grydeland H, Amlien I, Espeseth T, Reinvang I (2014). Accelerating cortical thinning: unique to dementia or universal in aging?. Cereb Cortex.

[7] Erickson KI, Gildengers AG, Butters MA (2013). Physical activity and brain plasticity in late adulthood. Dialogues Clin Neurosci.

[8] Batouli SAH, Trollor JN, Wen W, Sachdev PS (2014). The heritability of volumes of brain structures and its relationship to age: A review of twin and family studies. Ageing Res Rev.

[9] Fjell AM, Grydeland H, Krogsrud SK, Amlien I, Rohani DA, Ferschmann L (2015). Development and aging of cortical thickness correspond to genetic organization patterns. Proc Natl Acad Sci USA.

[10] Brouwer RM, Panizzon MS, Glahn DC, Hibar DP, Hua X, Jahanshad N (2017). Genetic influences on individual differences in longitudinal changes in global and subcortical brain volumes: Results of the ENIGMA plasticity working group. Hum Brain Mapp.

[11] Nyberg L, Boraxbekk CJ, Sörman DE, Hansson P, Herlitz A, Kauppi K (2020). Biological and environmental predictors of heterogeneity in neurocognitive ageing: evidence from Betula and other longitudinal studies. Ageing Res Rev.

[12] Cole JH, Marioni RE, Harris SE, Deary IJ (2019). Brain age and other bodily ‘ages’: implications for neuropsychiatry. Mol Psychiatry.

[13] Mather KA, Jorm AF, Parslow RA, Christensen H (2011). Is telomere length a biomarker of aging? A review. J Gerontol Ser A..

[14] Horvath S, Raj K (2018). DNA methylation-based biomarkers and the epigenetic clock theory of ageing. Nat Rev Genet.

[15] Elliott ML, Belsky DW, Knodt AR, Ireland D, Melzer TR, Poulton R (2021). Brain-age in midlife is associated with accelerated biological aging and cognitive decline in a longitudinal birth cohort. Mol Psychiatry.

[16] Cole JH, Franke K (2017). Predicting age using neuroimaging: innovative brain ageing biomarkers. Trends Neurosci.

[17] Kaufmann T, van der Meer D, Doan NT, Schwarz E, Lund MJ, Agartz I (2019). Common brain disorders are associated with heritable patterns of apparent aging of the brain. Nat Neurosci.

[18] Cole JH, Ritchie SJ, Bastin ME, Valdés Hernández MC, Muñoz Maniega S, Royle N (2018). Brain age predicts mortality. Mol Psychiatry.

[19] Cole JH, Poudel RPK, Tsagkrasoulis D, Caan MWA, Steves C, Spector TD (2017). Predicting brain age with deep learning from raw imaging data results in a reliable and heritable biomarker. NeuroImage..

[20] Fan CC, Brown TT, Bartsch H, Kuperman JM, Hagler DJ, Schork A (2017). Williams syndrome-specific neuroanatomical profile and its associations with behavioral features. NeuroImage Clin.

[21] Sun D, Ching CRK, Lin A, Forsyth JK, Kushan L, Vajdi A (2020). Large-scale mapping of cortical alterations in 22q11.2 deletion syndrome: Convergence with idiopathic psychosis and effects of deletion size. Mol Psychiatry.

[22] Sønderby IE, van der Meer D, Moreau C, Kaufmann T, Walters GB, Ellegaard M (2021). 1q21.1 distal copy number variants are associated with cerebral and cognitive alterations in humans. Transl Psychiatry.

[23] Sønderby IE, Gústafsson Ó, Doan NT, Hibar DP, Martin-Brevet S, Abdellaoui A (2020). Dose response of the 16p11.2 distal copy number variant on intracranial volume and basal ganglia. Mol Psychiatry.

[24] Silva AI, Ulfarsson MO, Stefansson H, Gustafsson O, Walters GB, Linden DEJ (2019). Reciprocal white matter changes associated with copy number variation at 15q11.2 BP1-BP2: a diffusion tensor imaging study. Biol Psychiatry.

[25] van der Meer D, Sønderby IE, Kaufmann T, Walters GB, Abdellaoui A, Writing Committee for the ENIGMA-CNV Working Group (2020). Association of copy number variation of the 15q11.2 BP1-BP2 region with cortical and subcortical morphology and cognition. JAMA Psychiatry.

[26] Chai JH, Locke DP, Greally JM, Knoll JHM, Ohta T, Dunai J (2003). Identification of four highly conserved genes between breakpoint hotspots BP1 and BP2 of the Prader-Willi/Angelman Syndromes deletion region that have undergone evolutionary transposition mediated by flanking duplicons. Am J Hum Genet.

[27] Rafi SK, Butler MG (2020). The 15q11.2 BP1-BP2 microdeletion (Burnside–Butler) syndrome: in silico analyses of the four coding genes reveal functional associations with neurodevelopmental disorders. Int J Mol Sci.

[28] Zwaag B, van der, Staal WG, Hochstenbach R, Poot M, Spierenburg HA, Jonge MVDE (2010). A co-segregating microduplication of chromosome 15q11.2 pinpoints two risk genes for autism spectrum disorder. Am J Med Genet B Neuropsychiatr Genet.

[29] De Rubeis S, Pasciuto E, Li KW, Fernández E, Di Marino D, Buzzi A (2013). CYFIP1 coordinates mRNA translation and cytoskeleton remodeling to ensure proper dendritic spine formation. Neuron..

[30] Oguro-Ando A, Rosensweig C, Herman E, Nishimura Y, Werling D, Bill BR (2015). Increased CYFIP1 dosage alters cellular and dendritic morphology and dysregulates mTOR. Mol Psychiatry.

[31] Silva AI, Haddon JE, Ahmed Syed Y, Trent S, Lin TCE, Patel Y (2019). Cyfip1 haploinsufficient rats show white matter changes, myelin thinning, abnormal oligodendrocytes and behavioural inflexibility. Nat Commun.

[32] Quamme GA (2010). Molecular identification of ancient and modern mammalian magnesium transporters. Am J Physiol-Cell Physiol.

[33] Murphy SM, Preble AM, Patel UK, O’Connell KL, Dias DP, Moritz M (2001). GCP5 and GCP6: two new members of the human γ-tubulin complex. Mol Biol Cell.

[34] Stefansson H, Rujescu D, Cichon S, Pietiläinen OPH, Ingason A, Steinberg S (2008). Large recurrent microdeletions associated with schizophrenia. Nature..

[35] Stefansson H, Meyer-Lindenberg A, Steinberg S, Magnusdottir B, Morgen K, Arnarsdottir S (2014). CNVs conferring risk of autism or schizophrenia affect cognition in controls. Nature..

[36] Kendall KM, Rees E, Escott-Price V, Einon M, Thomas R, Hewitt J (2017). Cognitive performance among carriers of pathogenic copy number variants: analysis of 152,000 UK Biobank subjects. Biol Psychiatry.

[37] Kendall KM, Bracher-Smith M, Fitzpatrick H, Lynham A, Rees E, Escott-Price V (2019). Cognitive performance and functional outcomes of carriers of pathogenic copy number variants: analysis of the UK Biobank. Br J Psychiatry.

[38] Salat DH, Buckner RL, Snyder AZ, Greve DN, Desikan RSR, Busa E (2004). Thinning of the cerebral cortex in aging. Cereb Cortex.

[39] Thambisetty M, Wan J, Carass A, An Y, Prince JL, Resnick SM (2010). Longitudinal changes in cortical thickness associated with normal aging. NeuroImage..

[40] Hogstrom LJ, Westlye LT, Walhovd KB, Fjell AM (2013). The structure of the cerebral cortex across adult life: age-related patterns of surface area, thickness, and gyrification. Cereb Cortex.

[41] Fjell AM, Westlye LT, Grydeland H, Amlien I, Espeseth T, Reinvang I (2013). Critical ages in the life course of the adult brain: nonlinear subcortical aging. Neurobiol Aging.

[42] Owen D, Bracher-Smith M, Kendall KM, Rees E, Einon M, Escott-Price V, et al. Effects of pathogenic CNVs on physical traits in participants of the UK Biobank. BMC Genomics. 2018;19. [cited 2021 Jun 21] Available from: https://www.ncbi.nlm.nih.gov/pmc/articles/PMC6278042/. 10.1186/s12864-018-5292-7 PMC6278042 30509170

[43] Chan MS, Arnold M, Offer A, Hammami I, Mafham M, Armitage J (2021). A biomarker-based biological age in UK Biobank: composition and prediction of mortality and hospital admissions. J Gerontol Ser A.

[44] Alfaro-Almagro F, Jenkinson M, Bangerter NK, Andersson JLR, Griffanti L, Douaud G (2018). Image processing and Quality Control for the first 10,000 brain imaging datasets from UK Biobank. NeuroImage..

[45] Fischl B (2012). FreeSurfer. NeuroImage..

[46] Glasser MF, Coalson TS, Robinson EC, Hacker CD, Harwell J, Yacoub E (2016). A multi-modal parcellation of human cerebral cortex. Nature..

[47] Chen T, Guestrin C. XGBoost: A Scalable Tree Boosting System. In: Proceedings of the 22nd ACM SIGKDD International Conference on Knowledge Discovery and Data Mining [Internet]. New York, NY, USA: Association for Computing Machinery; 2016. [cited 2022 Oct 3] p. 785–94. (KDD ’16). Available from: 10.1145/2939672.2939785.

[48] Chen T, He T, Benesty M, Khotilovich V, Tang Y, Cho H, et al. xgboost: Extreme Gradient Boosting. 2022. [cited 2022 Oct 4] Available from: https://CRAN.R-project.org/package=xgboost.

[49] Lange AMG, de, Kaufmann T, Meer D, van der, Maglanoc LA, Alnæs D, Moberget T (2019). Population-based neuroimaging reveals traces of childbirth in the maternal brain. Proc Natl Acad Sci USA.

[50] de Lange AMG, Anatürk M, Suri S, Kaufmann T, Cole JH, Griffanti L (2020). Multimodal brain-age prediction and cardiovascular risk: the Whitehall II MRI sub-study. NeuroImage..

[51] Le TT, Kuplicki RT, McKinney BA, Yeh HW, Thompson WK, Paulus MP (2018). A nonlinear simulation framework supports adjusting for age when analyzing brainAGE. Front Aging Neurosci.

[52] Smith SM, Vidaurre D, Alfaro-Almagro F, Nichols TE, Miller KL (2019). Estimation of brain age delta from brain imaging. NeuroImage..

[53] de Lange AMG, Cole JH. Commentary: Correction procedures in brain-age prediction. NeuroImage Clin. 2020 [cited 2021 Jan 28];26. Available from: https://www.ncbi.nlm.nih.gov/pmc/articles/PMC7049655/. 10.1016/j.nicl.2020.102229 PMC7049655 32120292

[54] Høgestøl EA, Kaufmann T, Nygaard GO, Beyer MK, Sowa P, Nordvik JE, et al. Cross-sectional and longitudinal MRI brain scans reveal accelerated brain aging in multiple sclerosis. Front Neurol. 2019 [cited 2021 Jun 22];10. Available from: 10.3389/fneur.2019.00450/full. 10.3389/fneur.2019.00450 PMC6503038 31114541

[55] Burnside RD, Pasion R, Mikhail FM, Carroll AJ, Robin NH, Youngs EL (2011). Microdeletion/microduplication of proximal 15q11.2 between BP1 and BP2: a susceptibility region for neurological dysfunction including developmental and language delay. Hum Genet.

[56] Fry A, Littlejohns TJ, Sudlow C, Doherty N, Adamska L, Sprosen T (2017). Comparison of sociodemographic and health-related characteristics of UK Biobank participants with those of the general population. Am J Epidemiol.

[57] Tyrrell J, Zheng J, Beaumont R, Hinton K, Richardson TG, Wood AR (2021). Genetic predictors of participation in optional components of UK Biobank. Nat Commun.

