# Supplementary material for: No signs of neurodegenerative effects in 15q11.2 BP1-BP2 copy number variant carriers in the UK Biobank
Source: Transl Psychiatry. 2023 Feb 18;13:61. doi: 10.1038/s41398-023-02358-w (PMC9938862; doi:10.1038/s41398-023-02358-w)
Supplement: Supplementary file 1 — Supplementary Materials [file 41398_2023_2358_MOESM1_ESM.docx]

**Supplementary information**

**Overview:**

**Supplementary note 1:** Participants

**Supplementary note 2:** Statistical power: sensitivity analysis

**Supplementary note 3:** Brain age prediction model using machine learning

**Supplementary note 4:** Correction method for the brain age models

**Supplementary note 5:** Model selection of age-related changes in motor, lung and heart function

**Supplementary note 6:** Motor, lung, and heart function and brain age

**Supplementary Table 1.** Descriptive statistics for deletion-carriers, deletion-controls, duplication-controls, duplication-carriers and the training sample.

**Supplementary Table 2.** Descriptive statistics for the control and multiple sclerosis groups

**Supplementary Table 3.** Deletion-carriers vs deletion control: Age-related changes in cortical thickness, surface area and subcortical volume

**Supplementary Table 4.** Duplication-carriers vs duplication control: Age-related changes in cortical thickness, surface area and subcortical volume

**Supplementary Table 5.** Brain age prediction in a sample of patients with multiple sclerosis

**Supplementary Table 6.** Final motor, lung, and heart function models from the model selection procedure: Deletion-carriers and deletion-controls

**Supplementary Table 7.** Final motor, lung, and heart function models from the model selection procedure: Duplication-carriers and duplication-controls.

**Supplementary Figures 1-3:** Group differences between deletion-carriers, deletion-controls, duplication-controls, and duplication carriers on measures of brain morphology

**Supplementary Figure 4:** Age-related changes between deletion-carriers and deletion-control and duplication and duplication-control in cortical thickness, surface area and subcortical volume.

**Supplementary Figure 5**. Correlations between chronological age and predicted age based on machine learning algorithm (both corrected and uncorrected).

**Supplementary Figures 6-9:** Group differences between deletion-carriers, deletion-controls, duplication-controls, and duplication carriers on measures of brain age gap

**Supplementary Figure 10.** Age-related changes in reaction time, grip strength, lung function, diastolic and systolic blood pressure in 15q11.2 BP1-BP2 CNV carriers versus matched controls.

**Supplementary Note 1: Participants**In total, 41,959 individuals with a set of T1-weighted MR images from the UK biobank and genetics were available to the study. Participants were excluded in quality control based on a Euler number exceeding 3 standard deviations (n = 717), missing Euler number (n = 3) or having cortical thickness, surface area or subcortical volume exceeding 4 SD after regressing out age, age^2^, sex, scanner site, affection status (i.e., diagnosed with a neurological disorder or mental/behavioral disorder), estimated intracranial volume (ICV) and Euler number (n = 88), leaving a total of 41,151 participants. CNV carriers were identified as previously described^1^. The final quality controlled imaging subset contained 124 15p11.2 BP1-BP2 deletion and 142 duplication carriers and 39,886 non-carriers (i.e. not carrying other pathogenic CNVs as per^1^). We extracted two controls groups for the deletion carriers and duplication carriers separately by matching each carrier on age, sex, scanner site, affection status (absence or presence of a reported psychiatric or neurological diagnosis as defined by a F or G-ICD10 diagnosis) and ICV using propensity scores with the “MatchIt” package in R ^2^. The remaining non-carrier sample was used as a training set for the machine learning (ML), following the removal of individuals with a reported psychiatric or neurological diagnosis as defined by a F or G-ICD10 diagnosis, leaving a total of 36,013 individuals (M_age_ = 64.4, SD_age_ = 7.56, 52.4% females). Descriptive statistics for the deletion-carriers, deletion-controls, duplication-controls, duplication-carriers and the training sample are presented in Supplementary Table 1.

As part of the validation of the brain age model prediction, we also extracted out individuals diagnosed with multiple sclerosis (ICD10-code: G35, n = 60) to compare against a healthy control group. Here, the deletion-control and duplication-control groups (excluding individuals with a F or G-ICD10 diagnosis) were used as the control group (n = 1210). Descriptive statistics for multiple sclerosis and control group are presented in Supplementary Table 2.

**Supplementary Note 2: Statistical Power: Sensitivity analysis**
We performed a power sensitivity analysis in G*power 3.1 to obtain the estimated effect size that can be reliably detected using an independent t-test given alpha level = .05 and power = .8. The estimated effect size was d = .28 for deletion carriers (n = 124) compared to deletion-control (n = 496), d = .26 for duplication (n = 142) to duplication-control (n = 568) and d = .35 for deletion carriers to duplication carriers. The reports from a previous study indicated thicker cortex (d = .36), lower surface area (d = -.41) and smaller nucleus accumbens (d = -.27) in deletion carriers compared to non-carriers^1^, which indicate that the current study has sufficient power to reliably detect differences in cortical thickness and surface area. We also expected that the smaller effects on subcortical volumes to accumulate for deletion carriers, yielding a larger effect size when using the total subcortical volume as dependent variable compared to the effect size estimated with nucleus accumbens alone. Thus, the current sample size should be sufficient to reliably detect differences in total subcortical volume as well.

**Supplementary note 3: Brain age prediction model using machine learning**
To tune the models, we ran a grid search on the parameters learning rate (i.e., 0.01, 0.05, 0.1) and maximum depth (i.e., 3,4, 5 6) with 50 iterations, ten-fold cross-validation and early stopping after 10 rounds (i.e., training stopped if the model did not improve within 10 rounds). Root mean squared error (RMSE) for the test set was used for early stopping. The initial prediction of the model was set to the mean age of the training group. Next, we used the parameters obtained from the models with the lowest RMSE value to train the ML model for the brain age prediction models with 5000 iterations as maximum and early stopping after 10 rounds.

**Supplementary note 4: Correction method for the brain age models**The corrected brain age models were based on the following corrected method where step 1 is conducted on the training group to obtain the slope and the intercept (where α is the slope, Ω represent the chronological age, and β is the intercept) ^3^:

1. Predicted age= α × Ω + β

Thus, the α and β are fixed numbers obtained from the training group and further used to correct for brain age bias in the test set:

2. Corrected predicted age = Predicted age+ [Ω − (α × Ω + β)]

Finally, the corrected predicted age is subtracted from the chronological age to obtain an estimate of the brain age gap.

3. Corrected brain age gap = Corrected predicted age – Ω

**Supplementary note 5: Model selection of age-related changes in motor, lung, and heart function**

We selected one cognitive measure and four biomedical measures that are associated with ageing^4^ and that have been associated with carrying a 15q11.2 BP1-BP2 CNV^1,5^ These measures included reaction time (Data-Field 20023), averaged hand grip strength for left and right hand (Data field 46 and 47), forced expiratory volume in 1-second (Data-Field 3063), systolic and diastolic blood pressure (Data filed 4079 and 4080) and body mass index (Data-Field 21001). Participants included in the analyses had measures from either one, two or three time points, thus analyses were conducted with mixed cross sectional and longitudinal data. To cope with the dependency in the data, we used a mixed effect model with a random effect of participant on intercepts with maximum likelihood as the estimator. To test the age-related changes in the abovementioned measures, we first tested three different models to capture the age model that best fitted the data with sex and affection status as covariates (including BMI for the blood pressure variables).

The models were as follows:
1) Y = Sex + Affection status + random (1 | participant ID) + error

2) Y = Age + Sex + Affection status + random (1 | participant ID) + error

3) Y = Age + Age^2^ + Sex + Affection status + random (1 | participant ID) + error

Secondly, to test whether 15q11.2 BP1-BP2 carriers deviated from their respective control group, two models were created with 1) an inclusion of a main term of carrier status and 2) an interaction term between age and carrier status to the age-model that best fitted the data (e.g., for a linear model):

4) Y = Age + Sex + Affection status + Carrier status + random (1 | participant ID) + error

5) Y = Age + Sex + Affection status + Carrier status + Age*Carrier status + random (1 | participant ID) + error

The models were tested step-wise using the Akaike Information Criterion (AIC), where the more complex model was selected if the AIC dropped with at least 2 and with a p-value < .05

**Supplementary note 6: Motor, heart and lung function and brain age**

Among the deletion-carriers and deletion-control group, there were no significant associations between brain age gap and reaction time (r (571) = .07, p = .092), grip strength (r (604) = -.08, p = 0.056), lung function (r (556) = -.07, p = .108) systolic (r (501) = .01, p = .906) and diastolic blood pressure (r (501) = .06, p = .215). Among the duplication-carriers and deletion-control group, there was a significant negative correlation between brain age gap and lung function (r (630) = -.17, p < .001), whereas there were no associations between brain age gap and reaction time (r (653) = .10, p =.012), grip strength (r (682) = -.08, p = .029), systolic (r (563) = .10, p =.0167) and diastolic blood pressure (r (563) = .09, p = .034).

References

1. Writing Committee for the ENIGMA-CNV Working Group *et al.* Association of Copy Number Variation of the 15q11.2 BP1-BP2 Region With Cortical and Subcortical Morphology and Cognition. *JAMA Psychiatry* **77**, 420–430 (2020).

2. Ho, D., Imai, K., King, G. & Stuart, E. A. MatchIt: Nonparametric Preprocessing for Parametric Causal Inference. *J. Stat. Softw.* **42**, 1–28 (2011).

3. de Lange, A.-M. G. & Cole, J. H. Commentary: Correction procedures in brain-age prediction. *NeuroImage Clin.* **26**, (2020).

4. Chan, M. S. *et al.* A Biomarker-based Biological Age in UK Biobank: Composition and Prediction of Mortality and Hospital Admissions. *J. Gerontol. Ser. A* **76**, 1295–1302 (2021).

5. Owen, D. *et al.* Effects of pathogenic CNVs on physical traits in participants of the UK Biobank. *BMC Genomics* **19**, (2018).

**Supplementary Table 1.** Descriptive statistics for deletion-carriers, deletion-controls, duplication-controls, duplication-carriers and the training sample.

|  | Deletion  (n = 124) | Deletion-control  (n = 496) | Duplication (n = 142) | Duplication-control  (n = 568) | Training set  (n = 36,013) |
| --- | --- | --- | --- | --- | --- |
| **Age**  Mean (SD)  Median [Min, Max] | 64.7 (7.5)  65.5 [49.3, 77.5] | 64.9 (7.4)  65.5 [47.2, 80.5] | 63.9 (7.5)  64.2 [46.7, 81.3] | 63.6 (7.82)  64.2 [48.3, 80.2] | 64.4 (7.6)  64.9 [44.9, 82.8] |
| **Sex**  Female | 62.0 (50.0%) | 248.0 (50.0%) | 75.0  (52.8%) | 300.0 (52.8%) | 18,880 (52.4%) |
| **Reported Diagnosis**      CNS/mental/behavioral  F-diagnosis  G-diagnosis  None | 9.0  (7.3%)  4.0  (3.2%)  5.0  (4.0%)  115 (92.7%) | 36.0  (7.3%)  17.0  (3.4%)  18.0  (3.6%)  460 (92.7%) | 15.0 (10.6%)  6.0  (4.2%)  9.0  (6.3%)  127  (89.4%) | 60.0  (10.6%)  28.0  (4.9%)  32.0  (5.6%)  508  (89.4%) | 0.0  (0%)  36, 013 (100%) |
| **Estimated ICV**  Mean (SD)  Median [Min, Max] | 1500000 (150000)  1500000  [1190000, 1900000] | 1500000 (154000)  1490000  [1120000, 1960000] | 1460000 (149000)  1430000  [1150000, 1890000] | 1460000 (149000)  1440000  [1110000, 1910000] | 14900000 (148000)  1480000  [722000, 2270000] |

Note. CNS/mental/behavioral = Central nervous system disease or mental/behavioral disorders. ICV = intracranial volume

**Supplementary Table 2.** Descriptive statistics for the control and multiple sclerosis groups

|  | Multiple Sclerosis  (n = 60) | Control-group (n = 1210) |
| --- | --- | --- |
| **Age**  Mean (SD)  Median [Min, Max] | 61.2 (7.86)  60.8 [47.9, 78.5] | 64.2 (7.59) 64.7 [47.2, 81.3] |
| **Sex**  Female | 43.0 (71.7%) | 650.0 (53.7%) |
| **Reported Diagnosis**  CNS/mental/behavioral  None | 60.0 (100%) 0.0 (0%) | 0.0 (0%) 1210 (100%) |
| **Estimated ICV**  Mean (SD)  Median [Min, Max] | 1450000 (135000) 1430000 [1170000,  1730000] | 1470000 (150000) 1460000 [1110000, 1960000] |

Note. CNS/mental/behavioral = Central nervous system disease or mental/behavioral disorders. ICV = intracranial volume

**Supplementary Table 3.** Deletion-carriers vs deletion-control: Age-related changes in cortical thickness, surface area and subcortical volume

|  | **Estimates** | **Std. Error** | **CI [95%]** | **Statistic** | **P-value** |
| --- | --- | --- | --- | --- | --- |
| **Cortical Thickness** |  |  |  |  |  |
| Intercept | -0.06 | 0.24 | -0.53,  0.40 | -0.27 | 0.789 |
| Age | 0.01 | 0.01 | -0.01,  0.02 | 0.89 | 0.372 |
| Age^2^ | -0.00 | 0.00 | -0.00,  0.00 | -1.49 | 0.136 |
| Deletion-carrier | 0.11 | 0.07 | -0.03,  0.25 | 1.53 | 0.126 |
| Age*Deletion-carrier | -0.00 | 0.00 | -0.00,  0.00 | -1.17 | 0.244 |
| **Surface Area** |  |  |  |  |  |
| Intercept | -29928.48 | 10684.15 | -50910.33,  -8946.62 | -2.80 | 0.005 |
| Age | 1065.69 | 334.46 | 408.87, 1722.50 | 3.19 | 0.002 |
| Age^2^ | -9.20 | 2.60 | -14.30,  -4.10 | -3.54 | <0.001 |
| Deletion-carrier | -3690.93 | 3299.86 | -10171.29, 2789.43 | -1.12 | 0.264 |
| Age*Deletion-carrier | 36.86 | 50.66 | -62.62,  136.34 | 0.73 | 0.467 |
| **Subcortical Volume** |  |  |  |  |  |
| Intercept | -7609.92 | 8536.68 | -24374.50, 9154.66 | -0.89 | 0.373 |
| Age | 415.96 | 267.23 | -108.84, 940.75 | 1.56 | 0.120 |
| Age^2^ | -4.58 | 2.07 | -8.65,  -0.50 | -2.21 | 0.028 |
| Deletion-carrier | 3498.01 | 2636.60 | -1679.82, 8675.85 | 1.33 | 0.185 |
| Age*Deletion-carrier | -69.97 | 40.48 | -149.45,  9.52 | -1.73 | 0.084 |

Note. Observations = 620, R^2^ = 0.165 (Cortical Thickness), 0.080 (Surface Area), 0.195 (Subcortical Volume) **Supplementary Table 4.** Duplication-carriers vs duplication-control: Age-related changes in cortical thickness, surface area and subcortical volume

|  | **Estimates** | **Std. Error** | **CI [95%]** | **Statistic** | **P-value** |
| --- | --- | --- | --- | --- | --- |
| **Cortical thickness** |  |  |  |  |  |
| Intercept | 0.04 | 0.20 | -0.36, 0.43 | 0.18 | 0.589 |
| Age | 0.00 | 0.01 | -0.01,  0.02 | 0.60 | 0.548 |
| Age^2^ | -0.00 | 0.00 | -0.00,  0.00 | -1.37 | 0.170 |
| Duplication-carrier | 0.01 | 0.07 | -0.12,  0.15 | 0.21 | 0.833 |
| Age*Duplication-carrier | -0.00 | 0.00 | -0.00,  0.00 | -0.32 | 0.752 |
| **Surface Area** |  |  |  |  |  |
| Intercept | -8145.15 | 9253.82 | -26313.50, 10023.20 | -0.88 | 0.379 |
| Age | 370.27 | 294.15 | -207.24, 947.78 | 1.26 | 0.209 |
| Age^2^ | -3.65 | 2.31 | -8.19,  0.89 | -1.58 | 0.115 |
| Duplication-carrier | 7146.67 | 3053.81 | 1151.01, 13142.32 | 2.34 | 0.020 |
| Age*Duplication-carrier | -125.45 | 47.52 | -218.74, -32.26 | -2.64 | 0.008 |
| **Subcortical Volume** |  |  |  |  |  |
| Intercept | -3304.56 | 7223.85 | -17487.40, 10878.28 | -0.46 | 0.647 |
| Age | 307.88 | 229.62 | -142.94, 758.71 | 1.34 | 0.180 |
| Age^2^ | -3.90 | 1.81 | -7.44,  -0.35 | -2.16 | 0.031 |
| Duplication-carrier | 3811.92 | 2383.91 | -868.50, 8492.34 | 1.60 | 0.110 |
| Age*Duplication-carrier | -58.03 | 37.09 | -130.86, 14.80 | -1.56 | 0.118 |

Note. Observations = 710, R^2^ = 0.181(Cortical Thickness), 0.067 (Surface Area), 0.215 (Subcortical Volume)

**Supplementary Table 6.** Brain age prediction in a sample of patients with multiple sclerosis

| Brain age model | Multiple Sclerosis | Healthy Controls | t-statistic (df) | p-value | Cohens d (CI) |
| --- | --- | --- | --- | --- | --- |
|  | Mean Brain Age (SD) | |  |  |  |
| Cortical thickness ML model | 2.55 (4.43) | -.13 (3.63) | 5.51 (1268) | < .001 | .73 (.47, .99) |
| Surface area ML model | 1.69 (4.10) | -.08 (3.60) | 3.69 (1268) | <.001 | .49 (.23, .75) |
| Subcortical volume ML model | 2.99 (4.47) | -.15 (3.81) | 6.16 (1268) | <.001 | .81 (.55, 1.08) |
| Full ML model | 3.75 (3.99) | -.19 (3.53) | 8.37 (1268) | <.001 | 1.11 (.84, 1.37) |

*Note.* SD = standard deviation, df = degrees of freedom, CI = 95% confidence interval. Mean brain age is adjusted for age, age^2^, sex, scanner site, intracranial volume, and Euler number. ML = machine learning **Supplementary Tables 7.** Final motor, heart and lung function models from the model selection procedure: Deletion-carriers and deletion-controls

|  | Estimates | Std.Error | CI (95%) | Statistic | P-value |
| --- | --- | --- | --- | --- | --- |
| **Reaction Time** |  |  |  |  |  |
| Intercept | 5.78 | 0.04 | 5.70, 5.86 | 147.48 | <0.001 |
| Age | 0.01 | 0.00 | 0.01, 0.01 | 17.26 | <0.001 |
| Sex [Male] | -0.03 | 0.01 | -0.06, -0.01 | -2.89 | 0.004 |
| Affection status [None] | -0.02 | 0.02 | -0.07, 0.02 | -1.04 | 0.298 |
| Deletion-carrier | 0.04 | 0.01 | 0.01, 0.07 | 2.85 | 0.004 |
| **Grip Strength** |  |  |  |  |  |
| Intercept | 38.47 | 1.65 | 35.24, 41.70 | 23.34 | <0.001 |
| Age | -0.27 | 0.02 | -0.31, -0.23 | -11.92 | <0.001 |
| Sex [Male] | 15.56 | 0.51 | 14.56, 16.57 | 30.42 | <0.001 |
| Affection status [None] | 1.65 | 0.99 | -0.28, 3.59 | 1.67 | 0.095 |
| **Lung Function** |  |  |  |  |  |
| Intercept | 4.41 | 0.13 | 4.16, 4.67 | 33.62 | <0.001 |
| Age | -0.04 | 0.00 | -0.04, -0.03 | -21.12 | <0.001 |
| Sex [Male] | 1.02 | 0.04 | 0.93, 1.10 | 23.27 | <0.001 |
| Affection Status [None] | 0.18 | 0.08 | 0.02, 0.35 | 2.21 | 0.028 |
| **Blood Pressure, Diastolic** |  |  |  |  |  |
| Intercept | 66.48 | 3.08 | 60.44, 72.52 | 21.58 | <0.001 |
| Age | -0.09 | 0.03 | -0.16, -0.03 | -2.87 | 0.004 |
| Sex [Male] | 3.57 | 0.68 | 2.23, 4.90 | 5.23 | <0.001 |
| Affection Status [None] | -0.27 | 1.31 | -2.83, 2.29 | -0.21 | 0.837 |
| BMI | 0.65 | 0.07 | 0.50, 0.79 | 8.76 | <0.001 |
| **Blood Pressure, Systolic** |  |  |  |  |  |
| Intercept | 76.15 | 5.80 | 64.77, 87.52 | 13.12 | <0.001 |
| Age | 0.63 | 0.06 | 0.51, 0.75 | 10.18 | <0.001 |
| Sex [Male] | 3.90 | 1.28 | 1.38, 6.41 | 3.04 | 0.002 |
| Affection Status [None] | 1.44 | 2.46 | -3.39, 6.26 | 0.58 | 0.560 |
| BMI | 0.78 | 0.14 | 0.51, 1.05 | 5.60 | <0.001 |

**Supplementary Tables 8.** Final motor, heart and lung function models from the model selection procedure: Duplication-carriers and duplication-control.

|  | Estimates | Std.Error | CI (95%) | Statistic | P-value |
| --- | --- | --- | --- | --- | --- |
| **Reaction Time** |  |  |  |  |  |
| Intercept | 5.76 | 0.03 | 5.70, 5.83 | 172.20 | <0.001 |
| Age | 0.01 | 0.00 | 0.01,0.01 | 19.21 | <0.001 |
| Sex [Male] | -0.04 | 0.01 | -0.06, -0.02 | -3.89 | <0.001 |
| Affection status [None] | 0.01 | 0.02 | -0.02, 0.05 | 0.84 | 0.402 |
| **Grip Strength** |  |  |  |  |  |
| Intercept | 42.15 | 1.37 | 39.47, 44.84 | 30.81 | <0.001 |
| Age | -0.32 | 0.02 | -0.35, -0.28 | -16.48 | <0.001 |
| Sex [Male] | 15.30 | 0.46 | 14.39, 16.20 | 33.04 | <0.001 |
| Affection status [None] | 0.68 | 0.75 | -0.80,2.16 | 0.90 | 0.367 |
| **Lung Function** |  |  |  |  |  |
| Intercept | 4.41 | 0.11 | 4.19, 4.63 | 39.08 | <0.001 |
| Age | -0.03 | 0.00 | -0.04, -0.03 | -21.88 | <0.001 |
| Sex [Male] | 0.95 | 0.04 | 0.88, 1.03 | 24.09 | <0.001 |
| Affection Status [None] | -0.01 | 0.06 | -0.14, 0.11 | -0.23 | 0.815 |
| **Blood Pressure, Diastolic** |  |  |  |  |  |
| Intercept | 66.35 | 2.93 | 60.61, 72.08 | 22.67 | <0.001 |
| Age | -0.08 | 0.03 | -0.15, -0.02 | -2.65 | 0.008 |
| Sex [Male] | 4.08 | 0.67 | 2.77, 5.39 | 6.10 | <0.001 |
| Affection Status [None] | -0.04 | 1.09 | -2.18, 2.09 | -0.04 | 0.968 |
| BMI | 0.63 | 0.07 | 0.50,0.77 | 9.11 | <0.001 |
| Duplication-carrier | 1.84 | 0.81 | 0.26, 3.41 | 2.28 | 0.023 |
| **Blood Pressure, Systolic** |  |  |  |  |  |
| Intercept | 105.69 | 16.03 | 74.30, 137.08 | 6.59 | <0.001 |
| Age | -0.44 | 0.54 | -1.50, 0.62 | -0.82 | 0.413 |
| Age^2^ | 0.01 | 0.00 | 0.00, 0.02 | 2.05 | 0.041 |
| Sex [Male] | 6.73 | 1.21 | 4.35,9.10 | 5.55 | <0.001 |
| Affection Status [None] | 0.80 | 1.97 | -3.06,4.66 | 0.40 | 0.686 |
| BMI | 0.80 | 0.12 | 0.55,1.04 | 6.40 | <0.001 |
| Duplication-carrier | 2.94 | 1.46 | 0.08,5.80 | 2.01 | 0.045 |


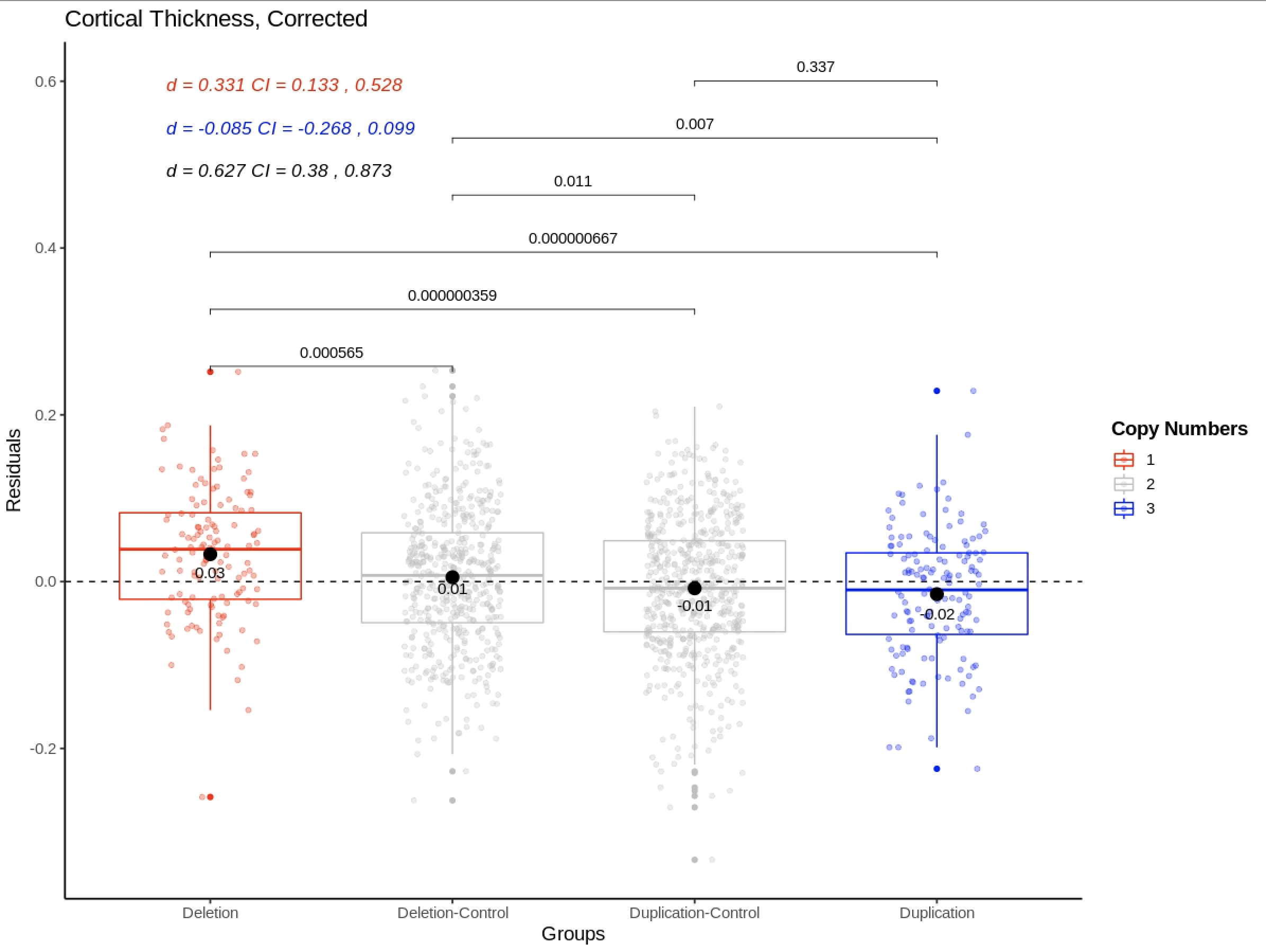

**Supplementary Figure 1.** Group differences in cortical thickness adjusted for age, age^2^, sex, scanner site, affection status, intracranial volume and Euler number. P-values are uncorrected and are based on two-sided independent t-tests. Cohens d are presented in the top left corner where the coloring of the effect sizes correspond to the following comparisons: Red = Deletion vs Deletion-Control, Blue = Duplication vs Duplication-Control, Black = Deletion vs Duplication. d = Cohens d, CI = 95% confidence interval.


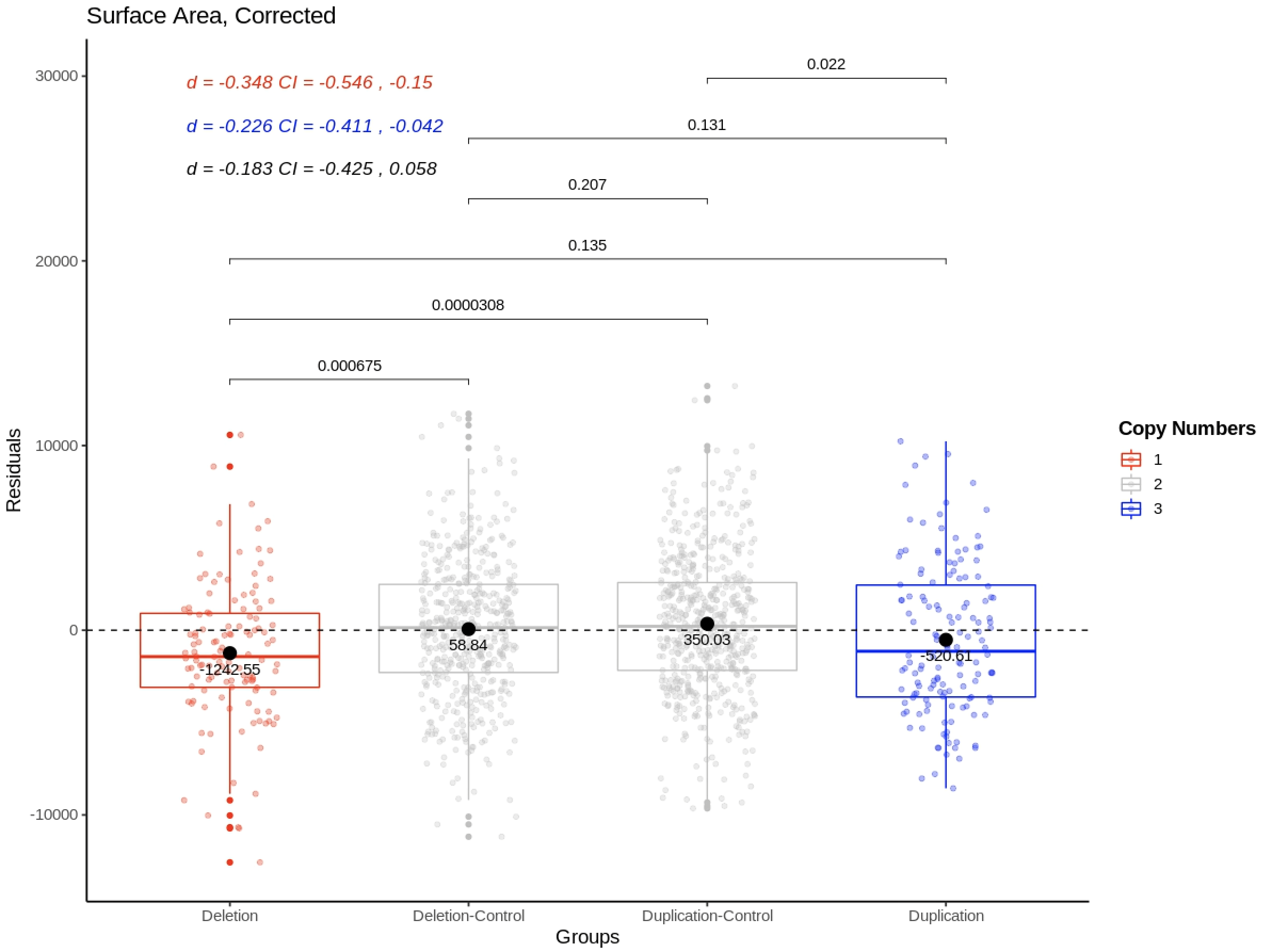

**Supplementary Figure 2.** Group differences in surface area adjusted for age, age^2^, sex, scanner site, affection status, scanner site, intracranial volume and Euler number. P-values are uncorrected and are based on two-sided independent t-tests. Cohens d are presented in the top left corner where the coloring of the effect sizes correspond to the following comparisons: Red = Deletion vs Deletion-Control, Blue = Duplication vs Duplication-Control, Black = Deletion vs Duplication. d = Cohens d, CI = 95% confidence interval.

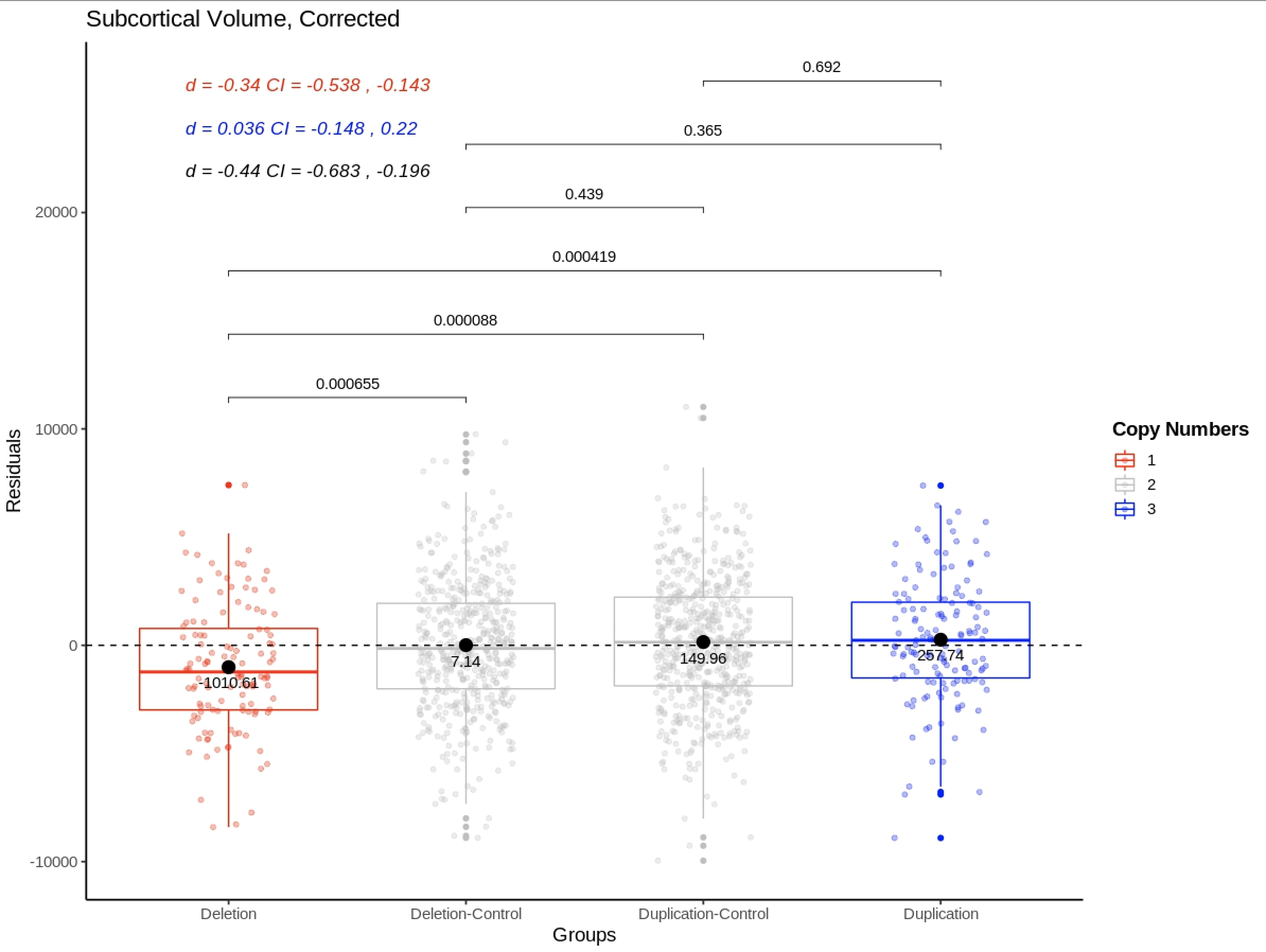

**Supplementary Figure 3.** Group differences in subcortical volume adjusted for age, age^2^, sex, scanner site, affection status, scanner site, intracranial volume and Euler number. P-values are uncorrected and are based on two-sided independent t-tests. Cohens d are presented in the top left corner where the coloring of the effect sizes correspond to the following comparisons: Red = Deletion vs Deletion-Control, Blue = Duplication vs Duplication-Control, Black = Deletion vs Duplication. d = Cohens d, CI = 95% confidence interval.

**
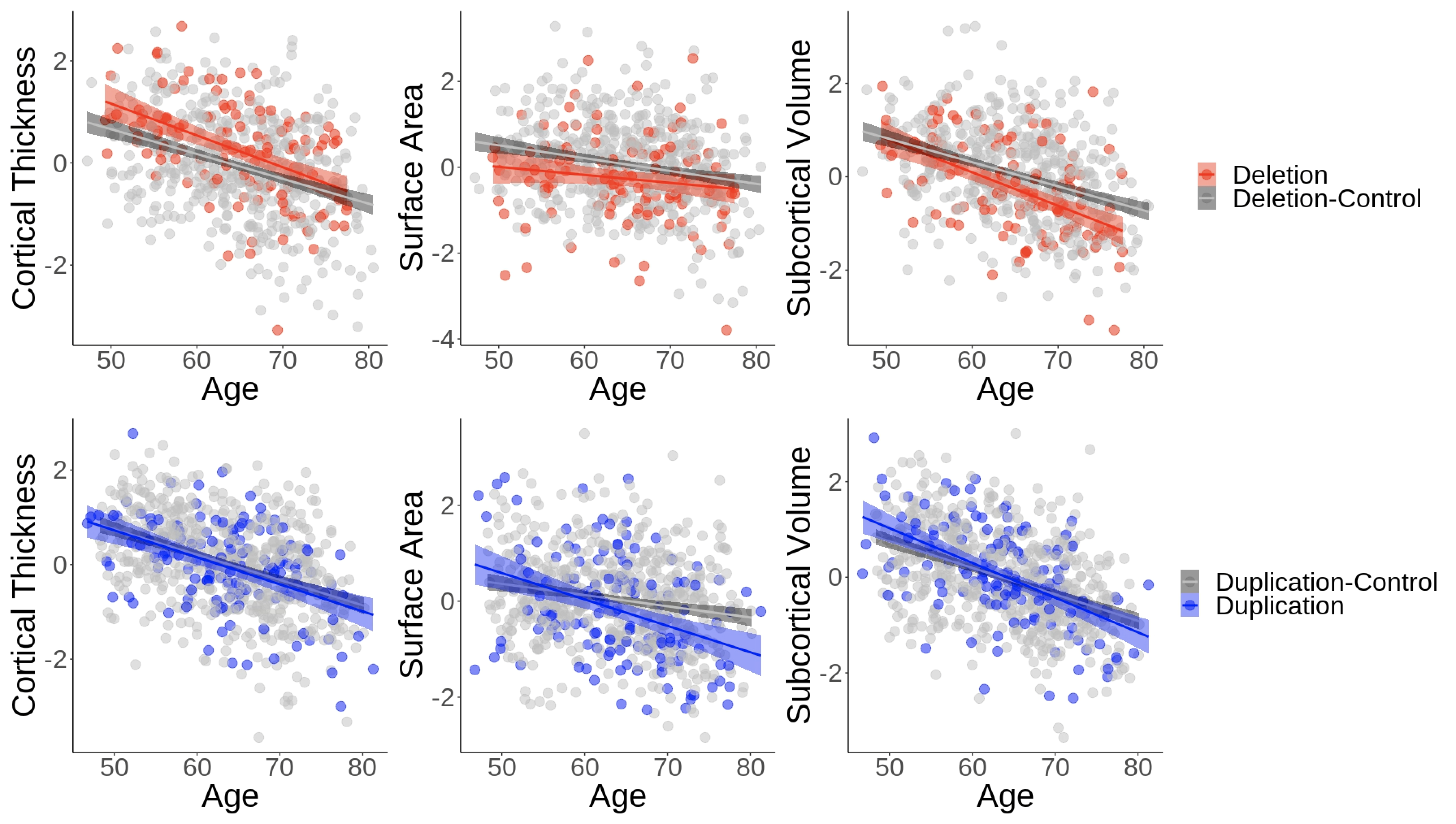

Supplementary Figure 4.** Age-related changes between deletion-carriers and deletion-controls (upper) and duplication-carriers and duplication-controls (lower) in cortical thickness, surface area and subcortical volume. Regression lines were fitted using linear regression, adjusting for sex, scanner site, affection status, intracranial volume and Euler number. The dependent variables were standardized for visualization purposes.


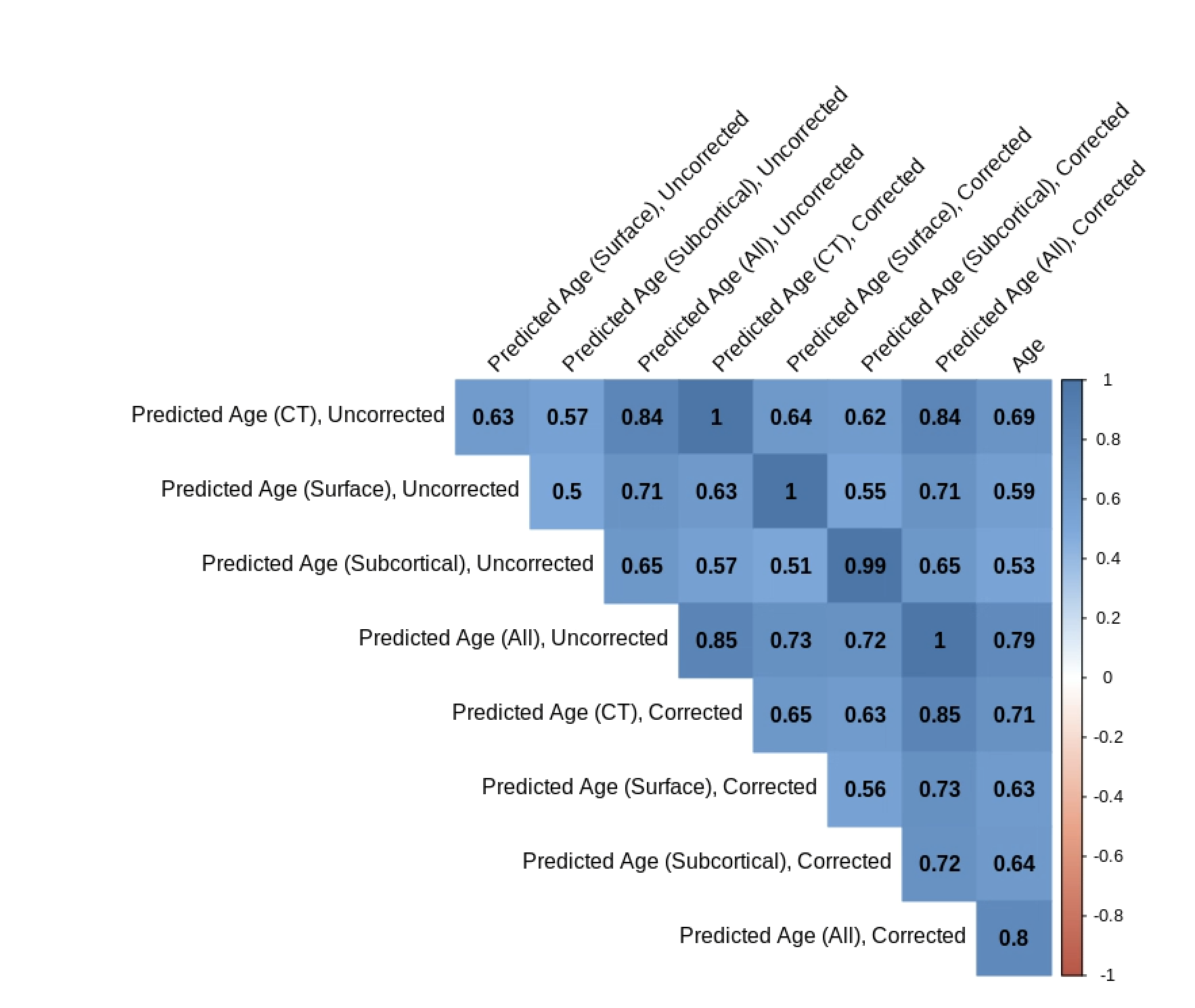


**Supplementary Figure 5**. Correlations between chronological age and predicted age based on machine learning algorithm (both corrected and uncorrected). CT = Cortical thickness only model, Surface = surface area only model, Subcortical = subcortical volume only model, All = full model.


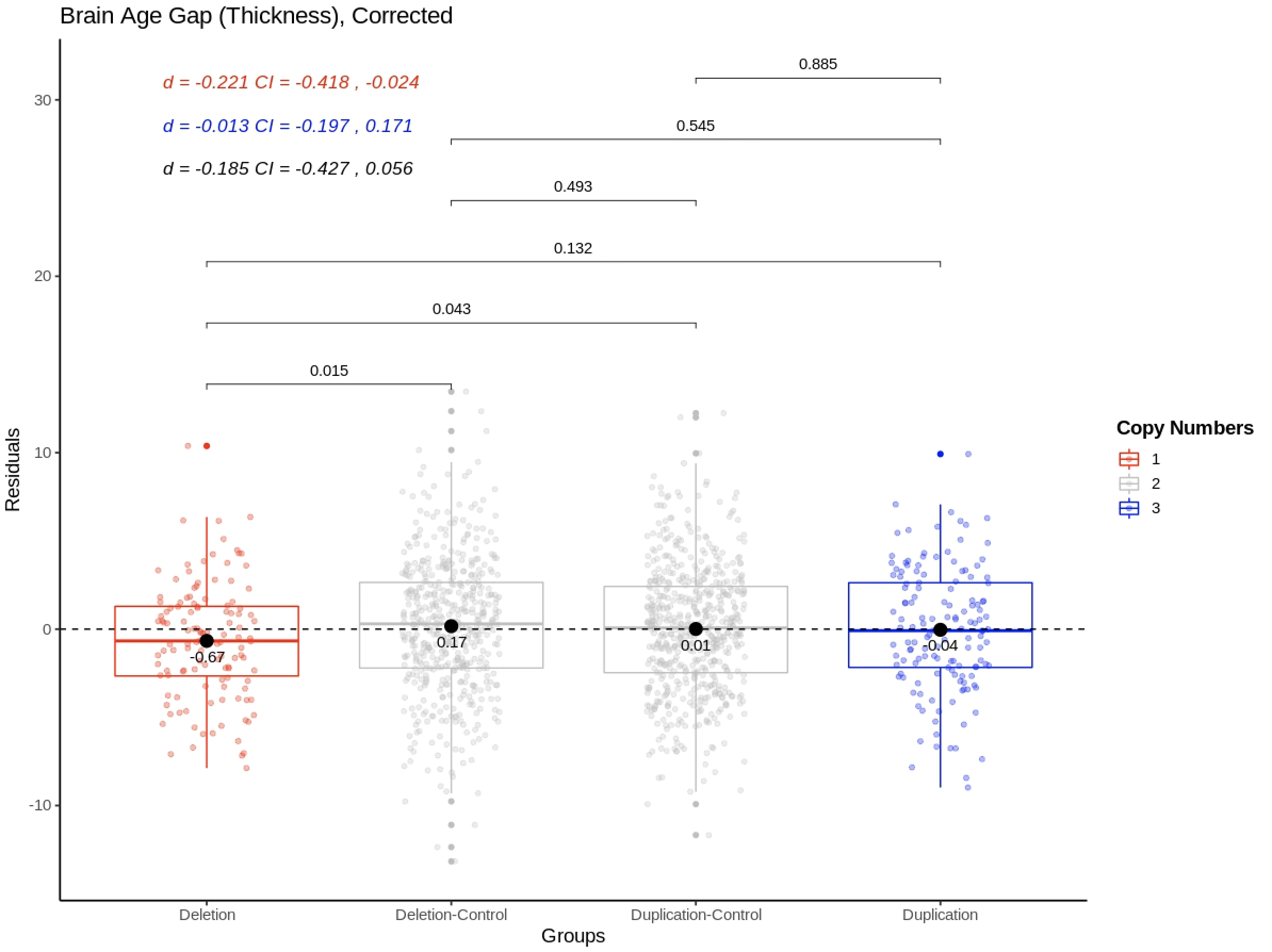

**Supplementary Figure 6.** Group differences in brain age gap, estimated through the cortical thickness only model, adjusted for age, age^2^, sex, scanner site, affection status, intracranial volume and Euler number. P-values are uncorrected and are based on two-sided independent t-tests. Cohens d are presented in the top left corner where the coloring of the effect sizes correspond to the following comparisons: Red = Deletion vs Deletion-Control, Blue = Duplication vs Duplication-Control, Black = Deletion vs Duplication. d = Cohens d, CI = 95% confidence interval.


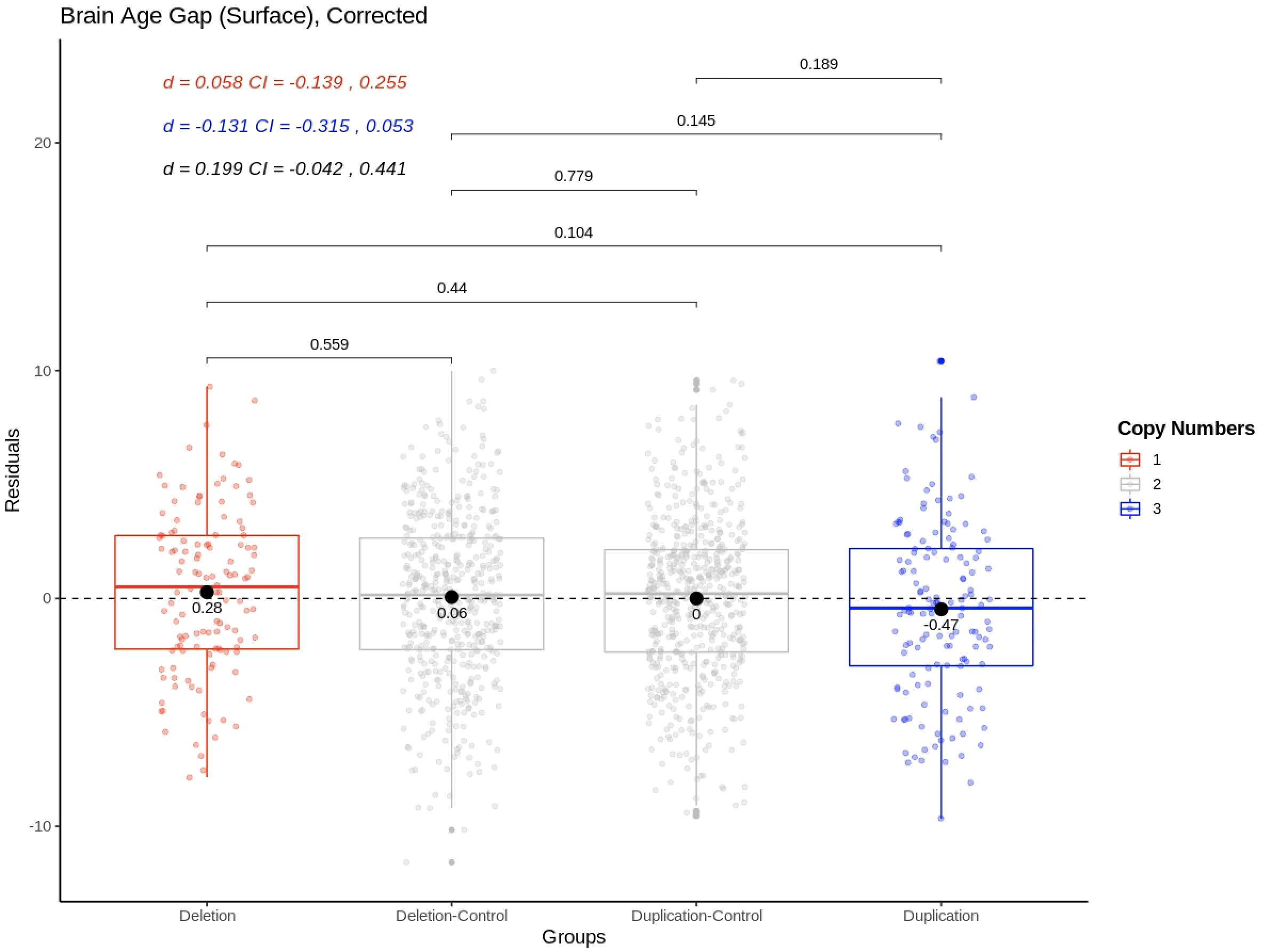

**Supplementary Figure 7.** Group differences in brain age gap, estimated through the surface area only model, adjusted for age, age^2^, sex, scanner site, affection status, intracranial volume and Euler number. P-values are uncorrected and are based on two-sided independent t-tests. Cohens d are presented in the top left corner where the coloring of the effect sizes correspond to the following comparisons: Red = Deletion vs Deletion-Control, Blue = Duplication vs Duplication-Control, Black = Deletion vs Duplication. d = Cohens d, CI = 95% confidence interval.


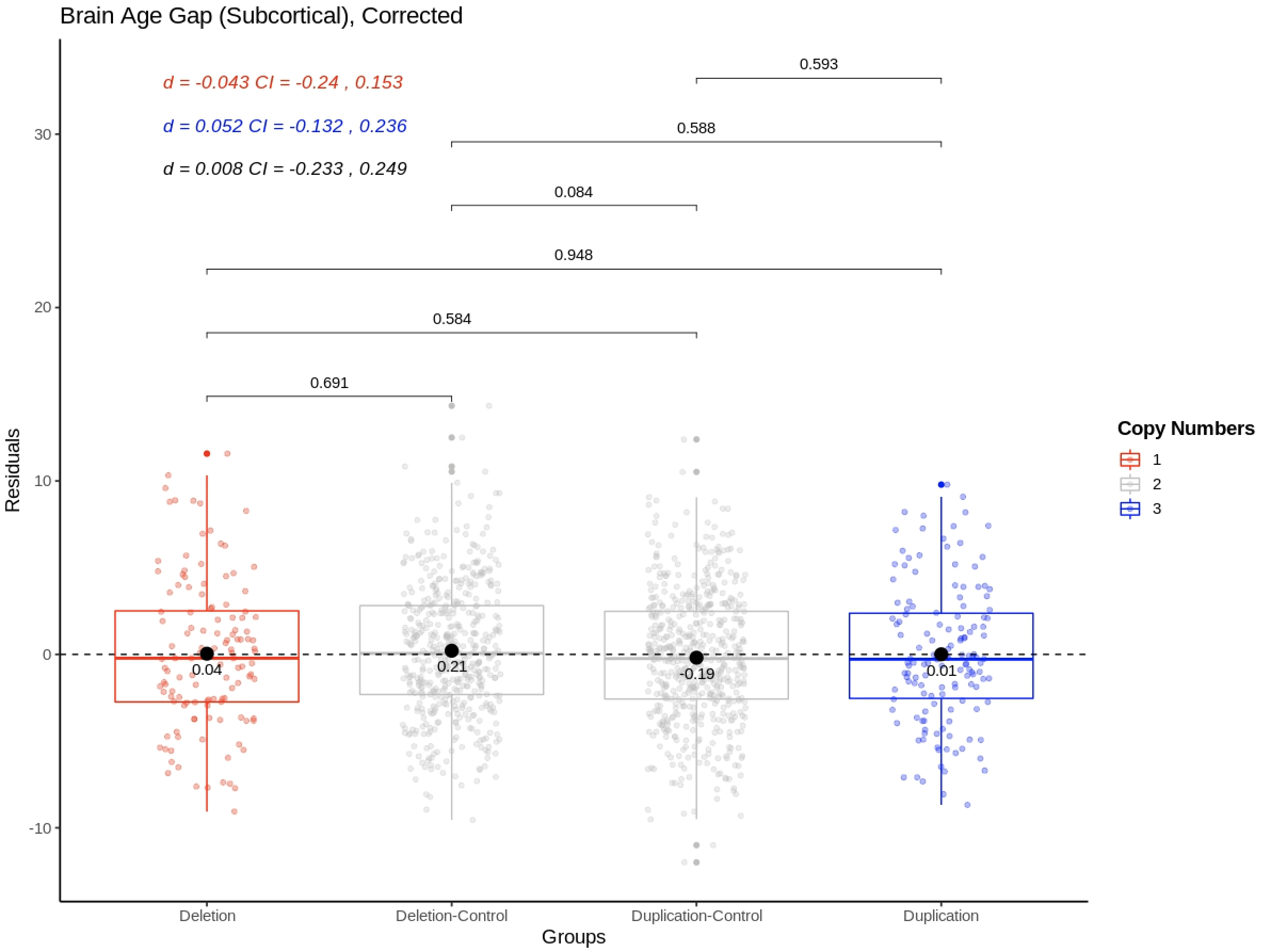

 **Supplementary Figure 8.** Group differences in brain age gap, estimated through the subcortical volume only model, adjusted for age, age^2^, sex, scanner site, affection status, intracranial volume and Euler number. P-values are uncorrected and are based on two-sided independent t-tests. Cohens d are presented in the top left corner where the coloring of the effect sizes correspond to the following comparisons: Red = Deletion vs Deletion-Control, Blue = Duplication vs Duplication-Control, Black = Deletion vs Duplication. d = Cohens d, CI = 95% confidence interval.


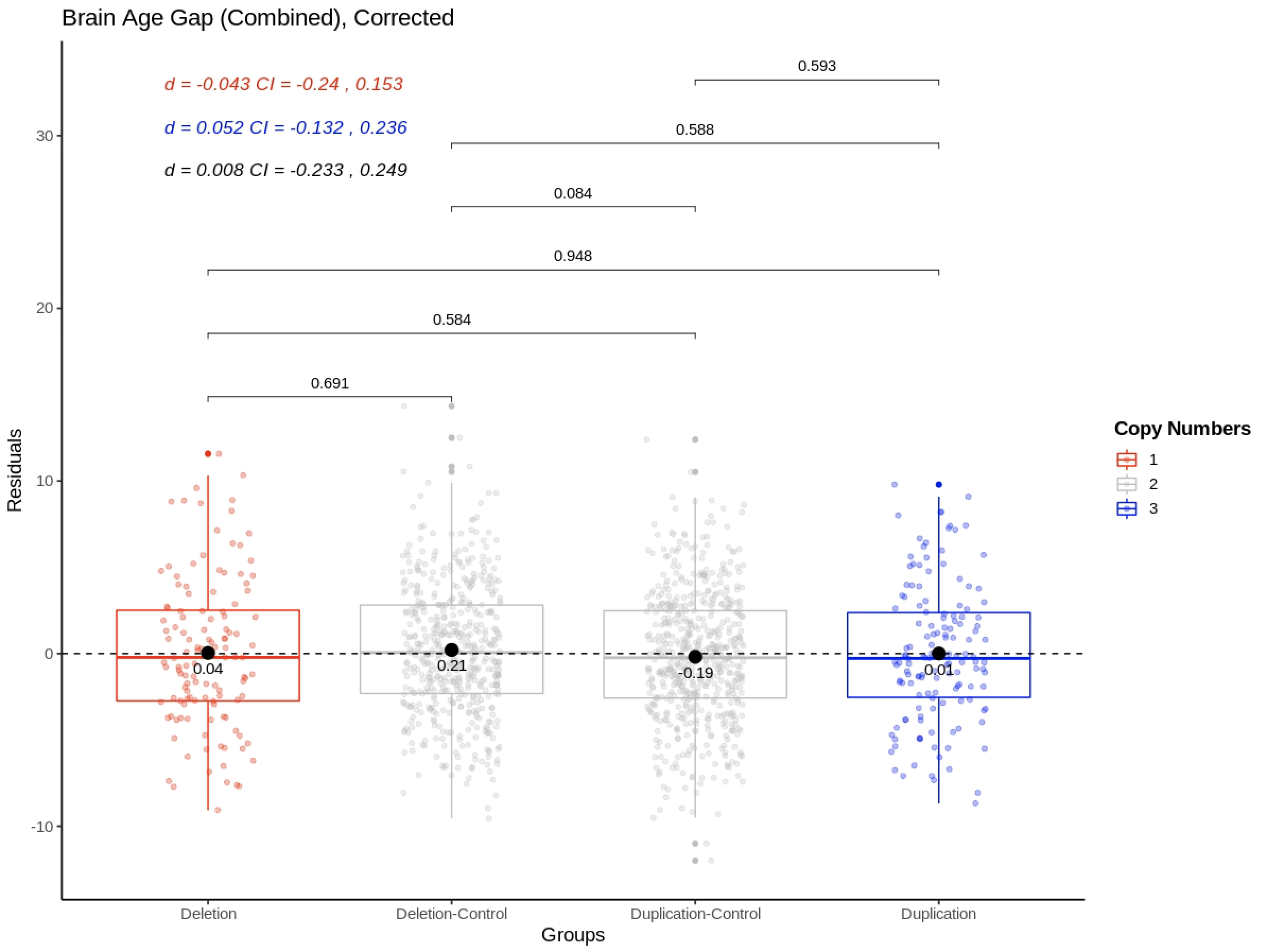


**Supplementary Figure 9.** Group differences in brain age gap, estimated through the full model, age, age^2^, sex, scanner site, affection status, intracranial volume and Euler number. P-values are uncorrected and are based on two-sided independent t-tests. Cohens d are presented in the top left corner where the coloring of the effect sizes correspond to the following comparisons: Red = Deletion vs Deletion-Control, Blue = Duplication vs Duplication-Control, Black = Deletion vs Duplication. d = Cohens d, CI = 95% confidence interval.

**
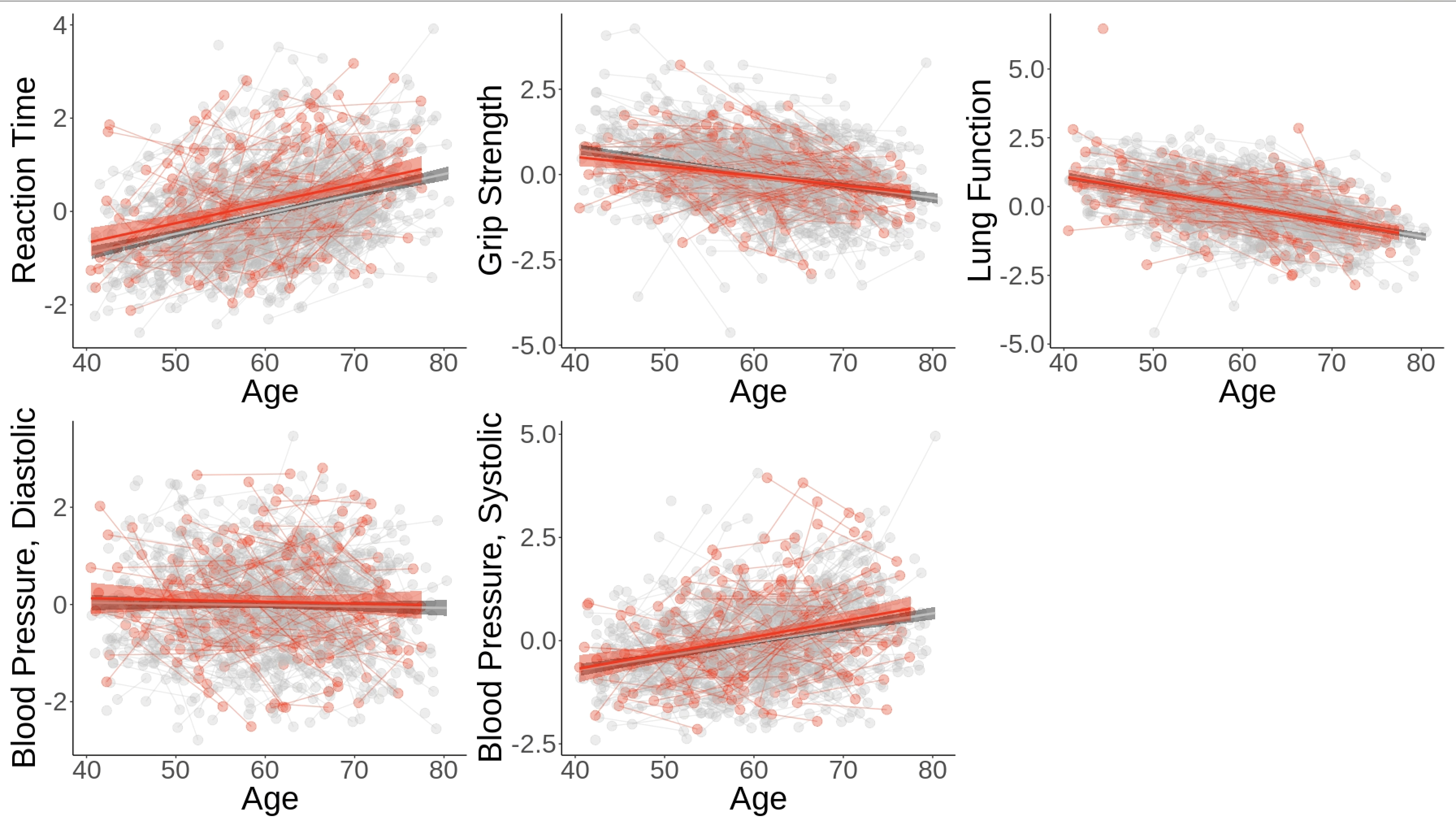

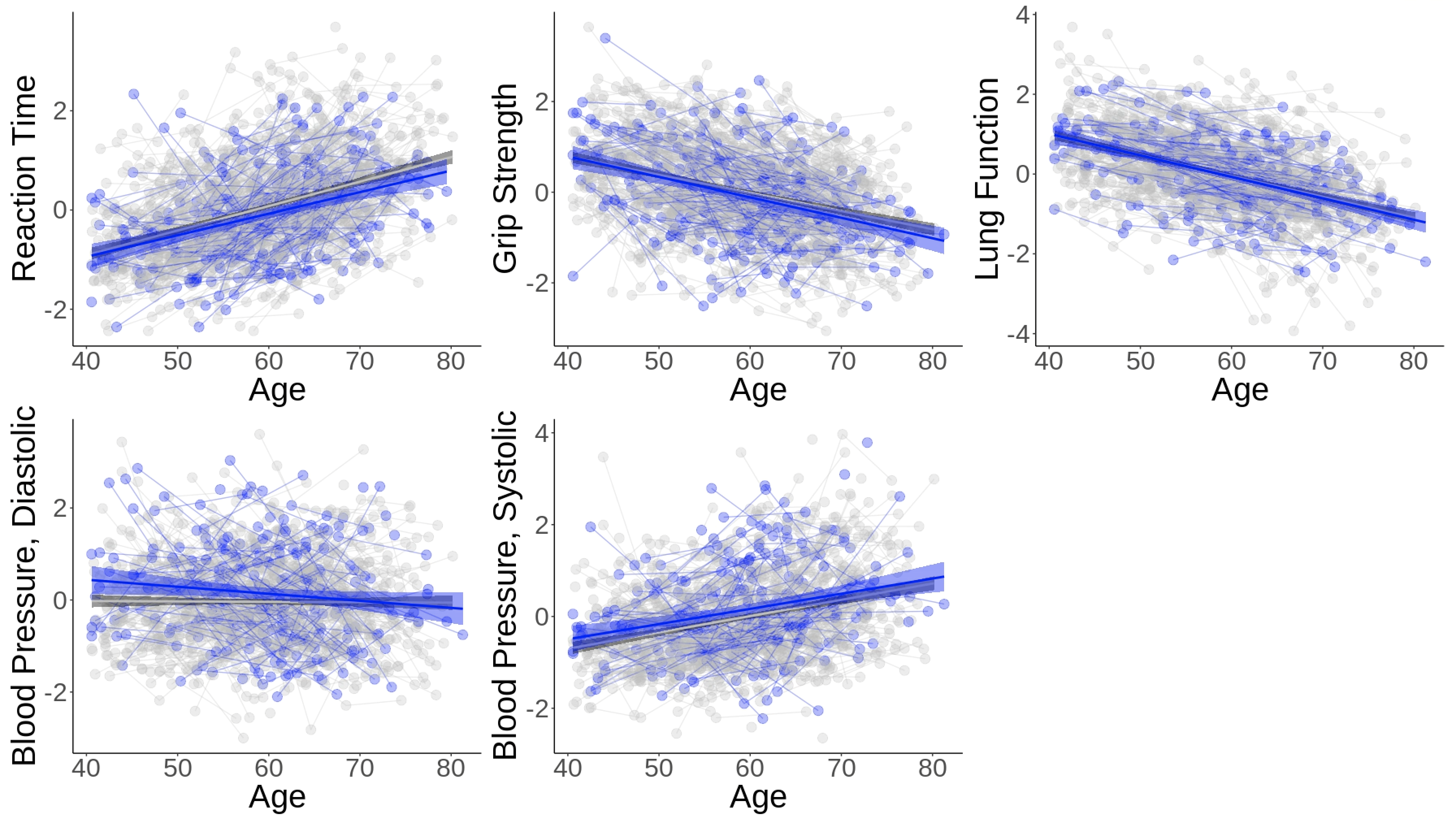
**

**Supplementary Figure 10.** Age-related changes in reaction time, grip strength, lung function, diastolic and systolic blood pressure in 15q11.2 BP1-BP2 CNV carriers versus matched controls. Red = deletion carriers, grey = non-carriers, blue = duplication carriers. All values were residualized for sex and affection status (incl. BMI for blood pressure) using linear regression and standardized for visualization purposes only. The results from the mixed effects models are shown in Supplementary Tables 7 (Deletion-carriers and deletion-controls) and Supplementary Tables 8 (Duplication-carriers and duplication-controls).
